# The divergent association of diet intake, parental education, and nutrition policy with childhood overweight and obesity from low- to high-income countries: A meta-analysis

**DOI:** 10.7189/jogh.14.04215

**Published:** 2024-11-22

**Authors:** Xue-Ting Liu, Yi-Di Wang, Yu-Jie Xu, Xiao-Yu Wang, Shu-Fang Shan, Jing-Yuan Xiong, Guo Cheng

**Affiliations:** 1 West China School of Public Health, West China Fourth Hospital, Sichuan University, Chengdu, China; 2 Laboratory of Molecular Translational Medicine, Centre for Translational Medicine, Key Laboratory of Birth Defects and Related Diseases of Women and Children, Sichuan University, Ministry of Education, Department of Paediatrics, West China Second University Hospital, Sichuan University, Chengdu, China

## Abstract

**Background:**

It is unclear whether the effects of dietary intake, parental education, and nutrition policy on childhood overweight and obesity is consistent between high-income (HICs) and low- and middle-income (LMICs) countries. The objective of this meta-analysis was to investigate the association of diet, parental education and nutrition policy with childhood overweight and obesity when the economic levels were controlled.

**Methods:**

PubMed, Web of Science, Embase and Scopus were searched for observational studies published from January 1980 to October 2023 that examined the association of diet, parental education and nutrition policy with childhood overweight and obesity. Meta random effects model stratified by gross national income *per capita* was used to assess whether the associations were varied by economic levels.

**Results:**

From 18 191 identified studies, 154 met the inclusion criteria. Meta-analysis revealed that higher sugar-sweetened beverage intake was a risk factor for childhood overweight and obesity in both HICs and LMICs countries/regions, whereas higher intake of fruit and/or vegetable was a protective factor only in LMICs countries/regions (odds ratio (OR) = 0.77; 95% confidence interval (CI) = 0.69–0.85). Moreover, lower parental education level increased the risk (OR = 1.46; 95% CI = 1.32–1.61) and nutrition policy implementation decreased the risk (OR = 0.96; 95% CI = 0.91–0.99) of childhood overweight and obesity only in HICs.

**Conclusions:**

Fruit and/or vegetable intake, parental education and nutritional policy exert different influences on childhood overweight and obesity in countries with varied economic levels. These findings will enhance the understanding of the complex interplay between these factors and their impact on childhood health.

Although the advancement of biomedical technology has significantly increased the life expectancy and quality [1], the different rates of national development have also led to growing global health disparities due to economic inequalities [2]. Global health disparities were evident as early as in childhood, in which the prevalence of childhood overweight and obesity is varied among countries with different levels of development [3], and the discrepancy might be also caused by other factors including environment and society. For instance, in Western countries such as countries in Europe, higher parental education level was associated with a lower prevalence of childhood overweight and obesity [4]; however, in most Asian countries, the opposite relationship is observed [5]. Being overweight and obese in childhood could lead to both short- [6–8] and long-term [9–11] negative health effects. Considering these adverse potential detrimental impacts, as well as the associated burden on the health care system and economy [12], it is crucial to address childhood overweight and obesity.

A conceptual framework of the social determinants of health, developed by the World Health Organization, offers a holistic perspective of the societal and environmental factors that impact health outcomes, which accounting for between 30–55% of health outcomes [13]. Among numerous determinants, nutrition-related factors may directly influence physiological functions and disease development, which are crucial for health promotion, especially childhood obesity prevention [14]. At the individual level, diet quality and quantity directly impact physiological functions and body weight, with poor dietary habits closely linked to the onset of chronic diseases [15]. At the family level, parental education influences health knowledge and child-rearing practices [16], and educated parents may foster healthier lifestyles in children [17]. At the national level, nutrition policies shape the overall food supply, determining food types and quality accessible to the population [18].

The effects of these determinants on childhood overweight and obesity are different among countries and the results of the prior research were also not consistent (vegetables and fruits [19,20], parental education [21–23], nutrition policies [24,25]), which might be due to different economic levels of these included studies. There is a complex relationship between these determinants of nutritional health and the economy. Economic factors have a significant impact on individuals’ dietary structure [26,27], the level of parental education [28,29], and nutrition-related policies [30], while on the other hand, education promotes economic growth [31], and nutritional policies may also influence economic development. To date, none of the previous studies of the determinants of childhood overweight or obesity took the role of the economy into account [19,20,23,32,33].

Therefore, this study aimed to examine the association of dietary intake, parental education, and nutrition policy with childhood overweight and obesity in terms of stratification of the country's economic status.

## METHODS

### Registration

This study was performed in accordance with the Preferred Reporting Items for Systematic Reviews and Meta-Analyses (PRISRMA) statement and Meta-analysis Of Observational Studies in Epidemiology (MOOSE) guideline (Table S1 in the **Online Supplementary Document**). The protocol for this review was registered with PROSPERO in November 2022 (registration number CRD42022366145).

### Search strategy

Two separate searches were conducted in PubMed, Web of Science, Embase, Scopus databases and other foreign databases, to identify observational studies investigating the association of dietary intake, prenatal education and nutrition policy with overweight and/or obesity among children published in January 1980 to November 2023. The search strategy was based on PICOS tool: P (Population) – toddlers and children, I (Intervention) – high dietary intakes, high parental education level and implementation of nutrition policy, C (Comparator) – low dietary intakes, low parental education level, no nutrition policy, O (Outcomes) – childhood overweight and/or obesity, S (Study type) – observational studies (detailed search strategy is available in Table S2 in the **Online Supplementary Document**). Moreover, the references of recent reviews and included studies were screened for additional references. The original data for abstracts and unpublished studies were obtained by emailing the corresponding authors. There were no language restrictions.

### Study selection and eligibility criteria

Studies were included if they met the following general criteria:

1) were cohort, case-control, cross-sectional studies or other types of observational studies

2) carried out in toddlers and children (2–18 years old)

3) reported frequency of children’s food consumption, parental educational level or nutrition policy (nutrition improvement programme at the national or regional level or school nutrition policy)

4) considered childhood overweight and/or obesity as an outcome variable.

Studies that considered only body mass index (BMI) or BMI z-score as an outcome variable were excluded. Given the heterogeneity in research questions, methods and outcomes of interest, intakes of fruit, vegetable and sugar-sweetened beverage were used as dietary intake indicators. More specifically, studies assessing either fresh or processed vegetables and fruits, or studies assessing any types of sugar-sweetened beverages (soft drinks, fruit drinks, energy drinks or sweetened milks) were included in the present study.

Studies were further selected if they fulfilled one of the following specific criteria:

1) compared children who had higher frequency of or liked to consume fruit, vegetable and sugar-sweetened beverages with their counterparts who consumed less, not consumed or disliked these foods

2) compared children whose parental education levels were lower secondary education or equivalent with their counterparts whose parental education levels were upper secondary education or higher

3) compared children who benefited from nutrition policy with demographically-similar counterparts who were not covered by policy, or compared children’s health outcomes before and after the policy implementation.

Studies were excluded if they were:

1) case reports, case series, reviews, and meta-analyses

2) carried out only in toddlers (<two years old) and children with certain diseases that could affect eating habits and/or physical development, such as immune diseases, severe infections and cardiovascular diseases

3) conducted in hospitalised patients.

Two reviewers (XTL, YDW) independently selected eligible studies (based on title and abstract, followed by full-text articles). Any disagreements were resolved through consensus or consultation with a senior reviewer (GC). Data extraction was conducted independently and in duplicate using a developed and standardised Excel-based digital extraction form. For each study, publication information, characteristics of study population, type of study design, intervention or exposure factors, outcomes and certainty of evidence assessment were extracted. The pooled estimate sizes, i.e. odds ratio (OR), risk ratio (RR), or hazard ratio (HR) with their 95% confidence interval (CI), were calculated.

### Assessment of risk of bias and certainty of evidence

Two researchers (XTL and YDW) independently assessed the certainty of all included studies with recommended tools [34]. Case-control, cohort studies and ecological studies were assessed by the Newcastle-Ottawa Scale tool, which was based on three broad perspectives, including the selection of the study groups, the comparability of the groups, and the ascertainment of either the exposure or outcome of interest for case-control or cohort studies respectively [35]. The Joanna Briggs Institute checklist was used to assess cross-sectional studies, and the checklist considered eight critical domains, including criteria for sample inclusion, detailed descriptions of study subjects and the setting, valid and reliable measurement for exposure and outcome, objective and standard measurement of condition, identification of confounding factors, strategies to deal with confounding factors, and appropriate statistical analysis [36]. Moreover, pre-post study was assessed by the National Institutes of Health quality assessment tool, which included 12 questions to access the risk of bias [37]. The checklist for each included study was assessed in duplicate.

### Economic status

According to the World Bank [38], the gross national income (GNI) *per capita* of each selected study was determined based on countries/regions and the time of project implementation: high-income countries/regions (GNI *per capita*>13 205 USD) and low- and middle-income countries/regions (GNI *per capita*>13 205 USD) [38] (Table S4 in the **Online Supplementary Document**). Considering that each province or city within a country may have different economic levels, the GNI *per capita* of each study was also determined based on economic level of the province or city that the study was conducted if that information was accessible. Within the high-income category or low- and middle-income category, studies were further stratified by countries/regions to perform sensitivity analysis.

### Statistical analyses

Statistical analysis was performed by STATA 15 (StataCorp LLC, College Station, TX, USA, 2017) and *R*, version 4.2.2. (R Core Team, Vienna, Austria, 2022). For each study, the exposure factors were re-categorised into dichotomous data if the factors were reported as multi-categorical. For dichotomous outcomes, we calculated pooled OR estimates and 95% CI.

To assess the mean effect sizes at the same economic level based on the World Bank Classification (GNI *per capita*), we assessed heterogeneity quantitatively using Cochran’s Q and *I*^2^ statistics for homogeneity within each subgroup. The fixed effects models were used to pool outcomes when significant heterogeneity was not present (*I^2^*<50%), and random effects models were used when significant heterogeneity was present (*I^2^*≥50%) [39]. *P*-values <0.05 were considered statistically significant. Subgroup analyses based on GNI *per capita*, and countries/regions were performed as sensitivity tests to assess the robustness of the result. Finally, Egger’s regression test were used to measure publication bias [40] (Figure S1 in the **Online Supplementary Document**).

## RESULTS

**Figure 1:**
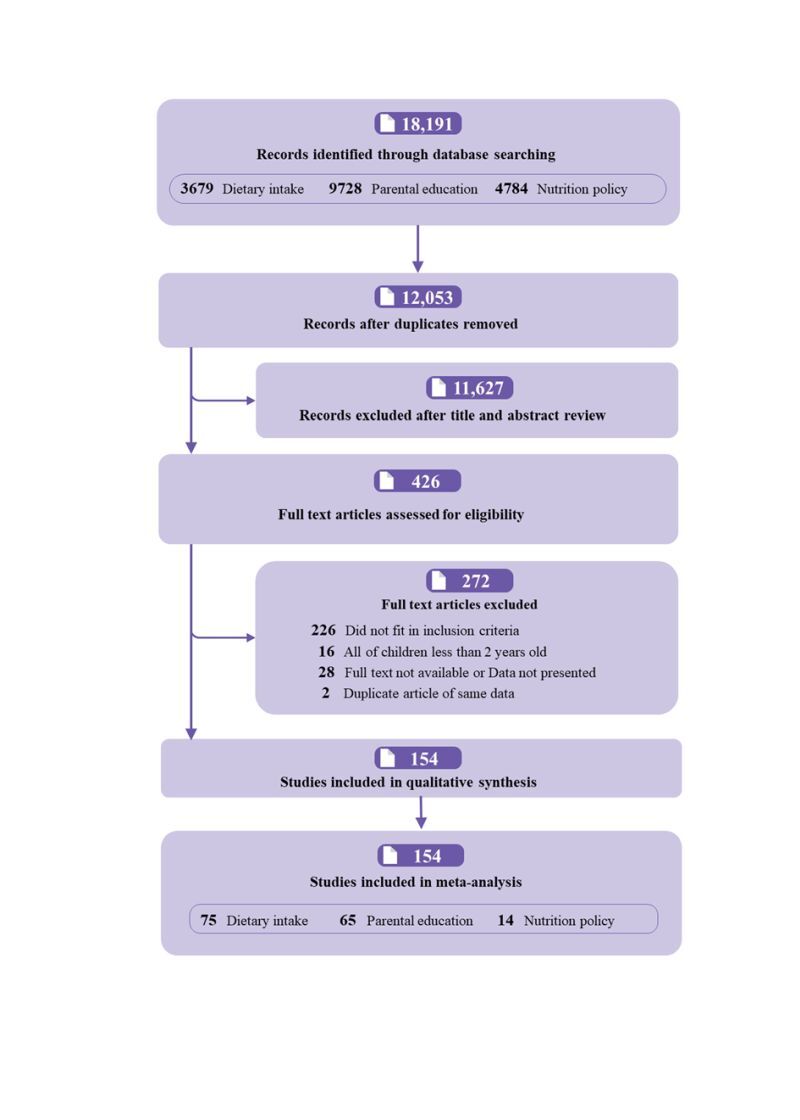
Flow diagram for selection of study.

The search of electronic databases identified 18 191 records with 12 053 articles remaining after the removal of duplicates. Of these, 11 900 articles were excluded, because these studies did not meet the selection criteria, leaving 154 articles included in the meta-analysis with a total sample size of n = 3 343 808 (**Figure 1**). Eighty-three of the included studies were conducted in Asia, 40 in Europe and 31 in America. Of these, most studies (81.8%) were cross-sectional studies, and over half of them (58.4%) were carried out in low- and middle-income countries/regions. There were 75, 65 and 14 articles investigating the association of dietary intake, prenatal education level, and nutrition policy with childhood overweight and obesity, respectively. For studies reporting dietary intake, all used food frequency questionnaires, but three studies used 24-hour dietary recalls. The detailed characteristics of each study are described in Table S3 in the **Online Supplementary Document**.

The assessment of risk of bias is shown in Table S3 in the **Online Supplementary Document**. Overall, the level of evidence for all included studies was adequate: 64.9% (n = 100) of the studies scored the highest level of certainty; in the remaining studies, 13.6% (n = 21) of the cross-sectional studies scored seven (maximum eight), 5.8% (n = 9) of the case-control, cohort and ecological studies scored eight (maximum nine), and 0.6% (n = 1) of the pre-post studies scored higher than nine (maximum 12). Some of the observational studies were rated as high risk because they did not adhere to the following critical domains:

1) did not identify confounding factors or mention the ways to address confounding factors (14 of 154, 9.1%)

2) were lack of comparability between exposed and unexposed groups in cohort study and pre-post study (five of 16, 31.3%).

Moreover, part of the studies (nine of 154, 5.8%) did not report funding sources or the inclusion criteria.

### Association of dietary intake with childhood overweight and obesity

**Figure 2:**
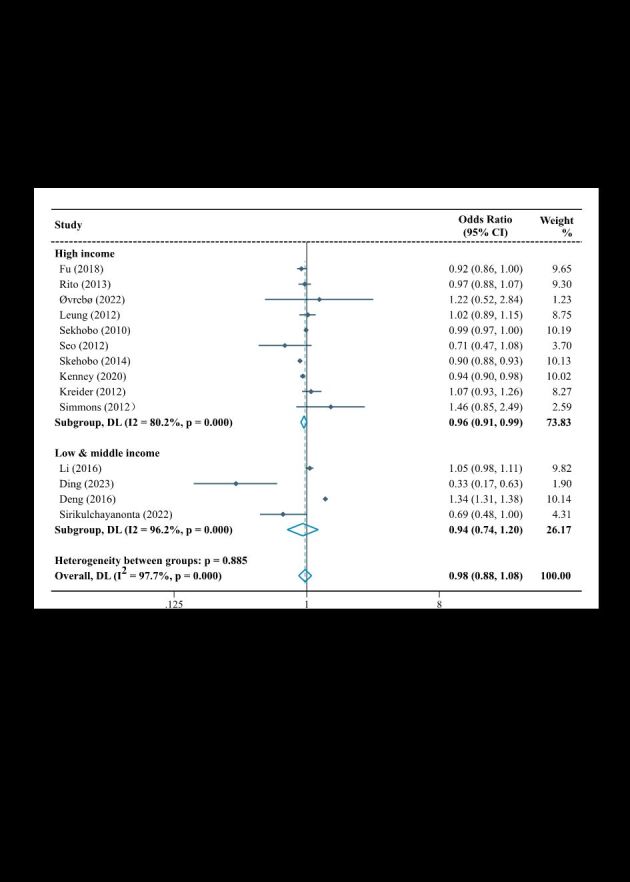
Forest plot of association between fruit and/or vegetable and overweight and/or obesity in children stratified by gross national income *per capita*. CI – confidence interval, DL – DerSimonian-Laird, OR – odds ratio.

Of 34 studies investigating the association of fruit and/or vegetable intake with overweight and obesity among children, nearly half of them investigated both vegetable and fruit intake (n = 15, 44.1%), and the majority were carried out in low- and middle-income countries/regions (n = 27, 79.4%). In the meta-analysis by GNI *per capita*, the pooled results showed that consuming more fruit and/or vegetable was a protective factor for childhood overweight and obesity in low- and middle-income countries/regions (OR = 0.77; 95% CI = 0.69–0.85), but not in high-income countries/regions (OR = 0.83; 95% CI = 0.68–1.02) (**Figure 2**).

**Figure 3:**
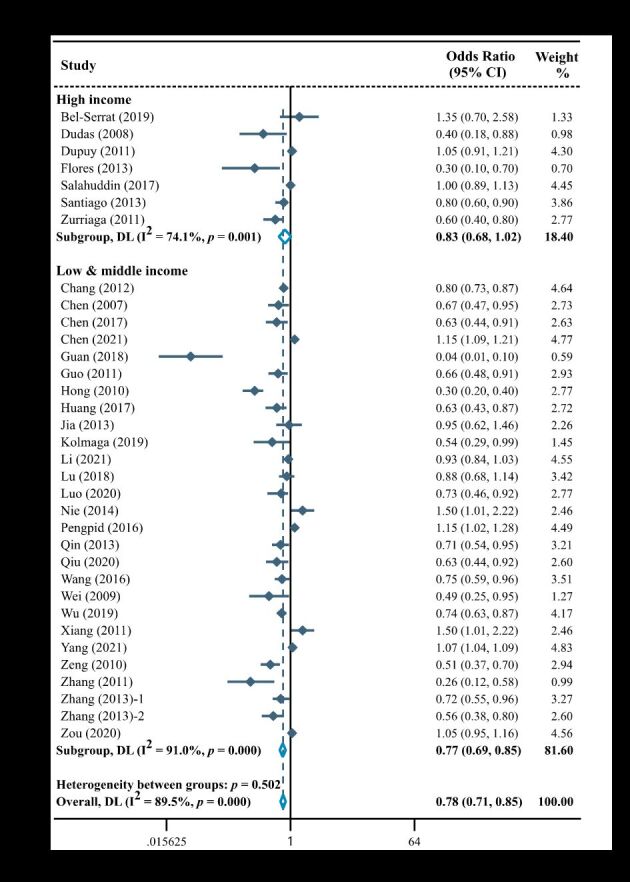
Forest plot of association between sugar-sweetened beverage intake and overweight and/or obesity in children stratified by gross national income *per capita.* CI – confidence interval, DL – DerSimonian-Laird, OR – odds ratio.

Fifty-one studies examined the relationship between sugar-sweetened beverage intake and childhood overweight and obesity. Among them, over half of studies were conducted in low- and middle-income countries/regions (n = 31, 60.8%). Meta-analysis by GNI *per capita* showed that consuming more sugar-sweetened beverages was a risk factor for childhood overweight and obesity in both low- and middle-income countries/regions (OR = 1.31; 95% CI = 1.21–1.41) and high-income countries/regions (OR = 1.24; 95% CI = 1.13–1.36) (**Figure 3**).

### Association of parental education level with childhood overweight and obesity

**Figure 4:**
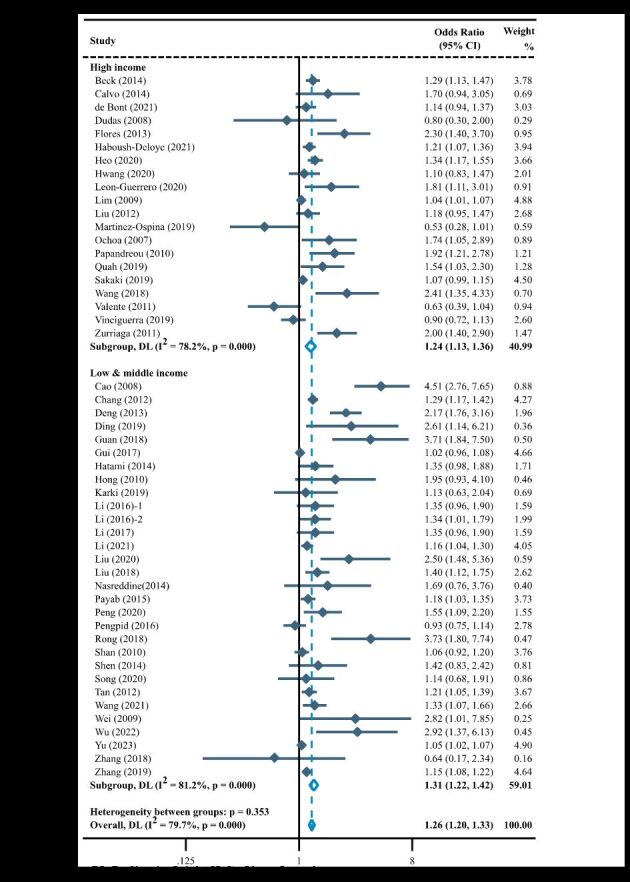
Forest plot of association between parental education level and overweight and/or obesity in children stratified by gross national income *per capita.* CI – confidence interval, DL – DerSimonian-Laird, OR – odds ratio.

A total of 65 studies investigating the association of parental education level with childhood overweight and obesity, and most studies were conducted in high-income countries/regions (n = 38, 58.5%). In the meta-analysis by GNI *per capita*, the pooled results showed that parental education level below junior high school was a risk factor for childhood overweight and obesity in high-income countries/regions (OR = 1.46; 95% CI = 1.32–1.61), but not in low- and middle-income countries/regions (OR = 1.04; 95% CI = 0.90–1.21) (**Figure 4**).

### Association of nutrition policy with childhood overweight and obesity

**Figure 5:**
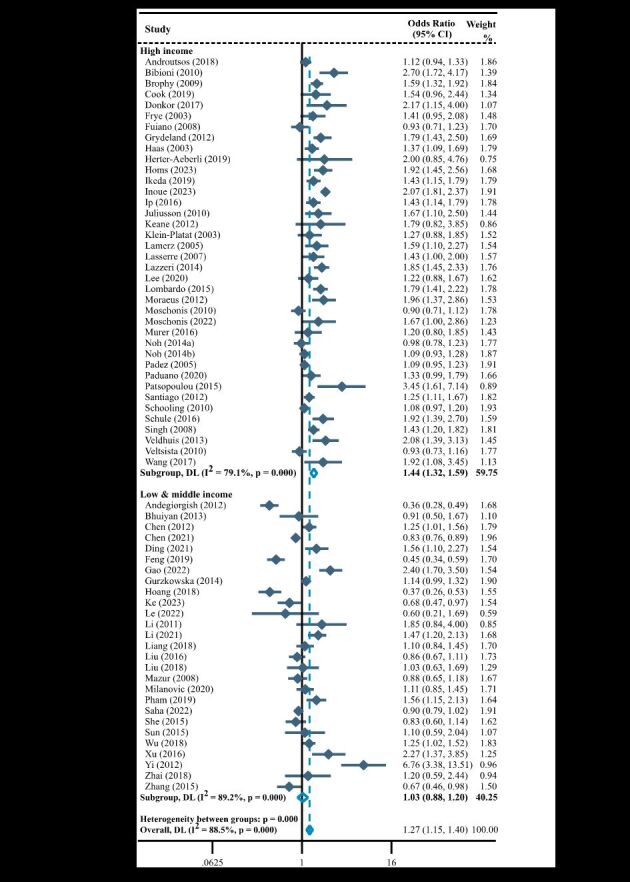
Forest plot of association between nutrition policy implementation and overweight and/or obesity in children stratified by gross national income *per capita.* CI – confidence interval, DL – DerSimonian-Laird, OR – odds ratio.

Of 14 studies examining the association of nutrition policy with childhood overweight and obesity, more than half of them (n = 10, 71.4%) were carried out in high-income countries/regions. The meta-analysis by GNI *per capita* showed that participation in nutrition policies was a protective factor for childhood overweight and obesity in high-income countries/regions (OR = 0.96; 95% CI = 0.91–0.99), but not in low- and middle-income countries/regions (OR = 0.94; 95% CI = 0.74–1.20) (**Figure 5**).

### Sensitivity analysis

Subgroup analyses based on GNI *per capita* and countries/regions were performed as sensitivity tests. The subgroup results were generally consistent with the results of meta-analysis by GNI *per capita* except for nutrition policy (Tables S5–6 in the **Online Supplementary Document**).

## DISCUSSION

The current study investigated the association of different levels of influencing factors with childhood overweight and obesity in countries with diverse economic statuses. The association of fruit and vegetables with childhood overweight and obesity was observed in low- and middle-income countries (LMICs), whereas the associations of parental education and nutritional policy were found only in high-income countries (HICs).

This study found that vegetable and fruit consumption acts as a protective factor against childhood overweight and obesity primarily in LMICs and this finding seems inconsistent with the previous study, which was conducted in both high and low-income countries [20]. High income countries, mainly situated in Western Europe and the USA, exhibit distinct dietary habits compared to most low-income Asian countries. Notably, Europe and the USA demonstrate relatively lower intakes of fresh vegetables and fruits [41], with a higher prevalence of canned alternatives [42]. These processed vegetables and fruits are abundant in sodium and added sugar [43], which may counteract the healthful effects of fresh produce, and the total caloric intake and portion size may be also a contributor. Moreover, sugar-sweetened beverage consumption increased the risk of childhood overweight and obesity in both HICs and LMICs in this study, which was consistent with the previous studies [20,32,33,44]. Sensitive analysis, however, revealed that in high-income European countries, no adverse association of sugar-sweetened beverage intake with childhood overweight and obesity was observed. The type of sugar-sweetened beverage intake in children is different among countries, with higher sugary dairy product intake among European children (e.g. The Healthy Lifestyle in Europe by Nutrition in Adolescence (HELENA) cohort) [45] but elevated soda consumption among children in the USA [46]. Yogurt and dairy beverages may pose a distinct obesity risk compared to other sugar-sweetened beverages. Components like milk fat [47], probiotics [48], and calcium [49] in dairy products have anti-inflammatory effects, can increase fat oxidation, and promote the feeling of satiety through cholecystokinin and glucagon-like peptide-1, thus acting as preventive measures against obesity. Dietary consumption, as well as other everyday living conditions, may have a direct impact on the health of children. It would be advisable to impose restrictions on the advertisement of sugar-sweetened and snacks, while concurrently encouraging the consumption of fresh vegetables and fruits.

Furthermore, the present study noticed that lower parental education was a significant risk factor for childhood overweight and obesity only in HICs, and this finding is consistent with the two systematic reviews conducted in developed countries [21,22]. This significant association between parental education and childhood obesity, however, was not observed in a prior systemic review by Wu et al., possibly due to the fact that the included studies were conducted in nations with varying economic levels [23]. The prevalence of childhood obesity was influenced by socioeconomic status, given that a higher prevalence of obesity was found in lower socioeconomic status families in developed countries [50] whereas a higher prevalence of obesity was observed in higher socioeconomic status families in developing countries [51]. During the past decades, many developing countries have experienced rapid economic development along with changes in lifestyle and diet, and this is evident in people living in urban areas with higher socioeconomic levels [52]. However, parents living in urban areas, albeit having a higher socioeconomic status including higher education level, still lack nutritional knowledge and awareness of a balanced diet and healthy body weight, and they may consider overweight and obesity as a symbol of affluence [23]. Moreover, HICs typically have well-functioning universal health care systems [53], and parental misconceptions or behaviours may be pointed out more quickly by professionals, allowing for timely identification and intervention for childhood obesity. In rural areas where there is a higher proportion of parents with lower education levels, food insecurity remains a major issue with malnourishment being the primary concern for child development rather than overweight and obesity [54]. These may partially explain why the inverse association of parental education and childhood overweight and obesity was not observed in LMICs. In these countries, it might be better to emphasise nutrition literacy education for parents and caregivers, as well as providing the necessary resources and support systems to increase access to healthy food.

Additionally, this study demonstrated that implementing nutrition policies was a protective factor for childhood overweight and obesity only in HICs, which was consistent with the previous systematic review that conducted in USA, Netherlands, UK and New Zealand [24]. The difference in the rates of childhood overweight and obesity between HICs and LMICs may partially account for it, given that the implementation of policies at the national level works well in countries/regions with higher rates of childhood overweight and obesity [3]. Moreover, low-income countries face the dual problems of malnutrition and over-nutrition, and nutrition policies in low-income countries have a greater effect on alleviating malnourishment and a lesser impact on childhood overweight and obesity, such as the ‘Nutrition Improvement Programme’ in China. Individuals, families, government and other stakeholder should all pay special attention to overweight and obesity prevention and management in children, particularly in these economically less developed countries. Countries at different economic levels may also be different in education [28,29], food industry [55], medical services [56,57], and nutrition-related industries [58,59], thus comprehensive and cross-sectoral approaches, involving individuals, families, governments, and other stakeholders are needed to prevent childhood overweight and obesity to some extent and leading to a more health-friendly society.

To explore the associations of determinants closely related to economy with childhood overweight and obesity at the individual, family, and societal levels, this study used subgroup analysis to control for economic factors. Another advantage lies in the inclusion of multiple databases during screening process, even grey literature. There are also some limitations in this study. At first, the high heterogeneity of the studies and the use of observational studies with varying study designs instead of randomised controlled trials may limit the generalisability of the results. Despite that, we mitigated this by using random effects models for the themes with high heterogeneity. Second, the number of studies on HICs under some themes is relatively small. Lastly, some studies had publication bias, which might affect the overall results.

## CONCLUSIONS

The consumption of fruits and vegetables may decrease the likelihood of children in LMICs being overweight or obese, while the influence of parental education and nutritional policies is only evident in HICs. The detrimental effects of beverages are observed across countries despite income levels. To effectively combat childhood overweight and obesity and foster a healthier society, a comprehensive and collaborative approach involving individuals, families, governments, and other stakeholders is necessary.

## Additional material


Online Supplementary Document
