# Supplementary material for: The divergent association of diet intake, parental education, and nutrition policy with childhood overweight and obesity from low- to high-income countries: A meta-analysis
Source: J Glob Health. 2024 Nov 22;14:04215. doi: 10.7189/jogh.14.04215 (PMC11586647; doi:10.7189/jogh.14.04215)
Supplement: Online Supplementary Document [file jogh-14-04215-s001.pdf]

Supplemental table 1: MOOSE Checklist.

Item	Recommendation	Reported on Page
Reporting of background should include		
1	Problem definition	6
2	Hypothesis statement	7
3	Description of study outcome(s)	8
4	Type of exposure or intervention used	7-8
5	Type of study designs used	7
6	Study population	7
Reporting of search strategy should include		
7	Qualifications of searchers (eg, librarians and investigators)	9
8	Search strategy, including time period included in the synthesis and key words	Supplemental table 2
9	Effort to include all available studies, including contact with authors	7
10	Databases and registries searched	7
11	Search software used, name and version, including special features used (eg, explosion)	8-9
12	Use of hand searching (eg, reference lists of obtained articles)	Supplemental table 2-3
13	List of citations located and those excluded, including justification	Supplemental table 2-3
14	Method of addressing articles published in languages other than English	7
15	Method of handling abstracts and unpublished studies	8
16	Description of any contact with authors	7
Reporting of methods should include		
17	Description of relevance or appropriateness of studies assembled for assessing the hypothesis to be tested	9
18	Rationale for the selection and coding of data (eg, sound clinical principles or convenience)	9
19	Documentation of how data were classified and coded (eg, multiple raters, blinding and interrater reliability)	9
20	Assessment of confounding (eg, comparability of cases and controls in studies wher	10
21	Assessment of study quality, including blinding of quality assessors, stratification or regression on possible predictors of study results	9
22	Assessment of heterogeneity	10-11
23	Description of statistical methods (eg, complete description of fixed or random effects models, justification of whether the chosen models account for predictors of study results, dose-response models, or cumulative meta-analysis) in sufficient detail to be replicated	10-11
24	Provision of appropriate tables and graphics	22-26
Reporting of results should include		
25	Graphic summarizing individual study estimates and overall estimate	23-26
26	Table giving descriptive information for each study included	Supplemental table 3
27	Results of sensitivity testing (eg, subgroup analysis)	Supplemental table 5
28	Indication of statistical uncertainty of findings	23-26
Reporting of discussion should include		
29	Quantitative assessment of bias (eg, publication bias)	Supplemental figure 2
30	Justification for exclusion (eg, exclusion of non-English language citations)	Supplemental table 5
31	Assessment of quality of included studies	Supplemental table 3
Reporting of conclusions should include		
32	Consideration of alternative explanations for observed results	14-17
33	Generalization of the conclusions (ie, appropriate for the data presented and within t	17
34	Guidelines for future research	14-17
35	Disclosure of funding source	14-17

Supplemental table 2. Search strategy to find potential relevant articles for inclusion in the meta-analysis

Topic	Database	Search terms	Retrieval
Dietary intake and risk of obesity and/or overweight	PubMed	((Dietary intake [Title/Abstract]) OR (Nutrient intake [Title/Abstract]) OR (Nutrients [MeSH Terms]) OR (Macronutrients [Title/Abstract]) OR (Energy intake [MeSH Terms]) OR (Protein intake [Title/Abstract]) OR (Fat intake [Title/Abstract]) OR (Carbohydrate intake [Title/Abstract]) OR (Food intake [Title/Abstract]) OR (Food groups [Title/Abstract]) OR (Vegetables [Title/Abstract]) OR (Fruit [Title/Abstract]) OR (Sugar sweetened beverage [Title/Abstract])) AND ((Body height [MeSH Terms]) OR (Body weight [MeSH Terms]) OR (Overweight [MeSH Terms]) OR (Obesity [MeSH Terms]) OR (Body Mass Index [MeSH Terms])) AND ((children [Title/Abstract]) OR (adolescent [Title/Abstract]) OR (student [Title/Abstract])) AND ((Obeservational study [Text Word]) OR (Cross-sectional [Text Word]) OR (Case-control [Text Word]) OR (Cohort [Text Word]) OR (Prospective [Text Word]) OR (Longitudinal [Text Word]))	409
	Web of Science	((AB=(Dietary intake)) OR AB=(Nutrient intake)) OR TS=(Nutrients)) OR AB=(Macronutrients)) OR TS=(Energy intake)) OR AB=(Protein intake)) OR AB=(Fat intake)) OR AB=(Carbohydrate intake)) OR AB=(Food intake)) OR AB=(Food groups)) OR AB=(Vegetables)) OR AB=(Fruit)) OR AB=(Sugar sweetened beverage))AND ((TS=(Body height)) OR TS=(Body weight)) OR TS=(Overweight)) OR TS=(Obesity)) OR TS=(Body Mass Index)) AND ((AB=(children) OR AB=(adolescent) OR AB=(student)) AND (AB=(Obeservational study) OR AB=(Cross-sectional) OR AB=(Case-control) OR AB=(Cohort) OR AB=(Prospective) OR AB=(Longitudinal))	1044
	Embase	(Dietary intake or Nutrient intake or Nutrients or Macronutrients or Energy intake or Protein intake or Fat intake or Carbohydrate intake or Food intake or Food groups or Vegetables or Fruit or Sugar sweetened beverage).mp. and (Body height or Body weight or Overweight or Obesity or Body Mass Index).mp. and (children or adolescent or student).mp. and (Obeservational study or Cross-sectional or Case-control or Cohort or Prospective or Longitudinal).mp.	141
	Scopus	(TITLE-ABS-KEY (Dietary intake OR Nutrient intake OR Nutrients OR Macronutrients OR Energy intake OR Protein intake OR Fat intake OR Carbohydrate intake OR Food intake OR Food groups OR Vegetables OR Fruit OR Sugar sweetened beverage) AND TITLE-ABS-KEY (Body OR Height OR Body weight OR Overweight OR Obesity OR Body mass index) AND TITLE-ABS-KEY (Children OR Adolescent OR student)) AND TITLE-ABS-KEY (Obeservational study or Cross-sectional or Case-control or Cohort or Prospective or Longitudinal)	854
	CNKI	(Title/Keyword/Abstract=dietary + nutrient + macronutrient + diet + energy intake + protein intake + fat intake + carbohydrate intake + food group + vegetable intake + fruit intake + beverage intake) AND (Title/Keyword/Abstract=body weight + height + obesity + overweight + body mass intake) AND (Keyword =children + adolescent + student) AND (Text Word = observational + cross-sectional + case-control + cohort +prospective)	1231

Supplemental table 2. Search strategy to find potential relevant articles for inclusion in the meta-analysis

Topic	Database	Search terms	Retrieval
Parental education level and risk of obesity and/or overweight	PubMed	((Educational level [Title/Abstract]) OR (Maternal education [Title/Abstract]) OR (Parental education [Title/Abstract])OR (Effect of education [Title/Abstract]) OR (Parental characteristics [Title/Abstract]) OR (Socioeconomic status [Title/Abstract]) OR (Social determinant [Title/Abstract])) AND ((Body height [MeSH Terms]) OR (Body weight [MeSH Terms]) OR (Overweight [MeSH Terms]) OR (Obesity [MeSH Terms])) AND ((children [Title/Abstract]) OR (adolescent [Title/Abstract]) OR (student [Title/Abstract])) AND ((Obeservational study [Text Word]) OR (Cross-sectional [Text Word]) OR (Case-control [Text Word]) OR (Cohort [Text Word]) OR (Prospective [Text Word]) OR (Longitudinal [Text Word]))	4086
	Web of Science	((AB=(Educational level)) OR AB=(Maternal education)) OR TS=(Parental education) OR AB=(Socioeconomic status) OR TS=(Energy intake)) OR AB=(Protein intake)) OR AB=(Fat intake)) OR AB=(Carbohydrate intake)) OR AB=(Food intake)) OR AB=(Food groups)) OR AB=(Vegetables)) OR AB=(Fruit)) OR AB=(Sugar sweetened beverage))AND ((TS=(Body height)) OR TS=(Body weight)) OR TS=(Overweight)) OR TS=(Obesity)) OR TS=(Body Mass Index)) AND ((AB=(children) OR AB=(adolescent) OR AB=(student)) AND (AB=(Obeservational study) OR AB=(Cross-sectional) OR AB=(Case-control) OR AB=(Cohort) OR AB=(Prospective) OR AB=(Longitudinal))	1372
	Embase	(Educational level or Maternal education or Parental education or Effect of education or Parental characteristics or Socioeconomic status or Social determinant). ab. and (Body height or Body weight or Overweight or Obesity or Body Mass Index). ab. and(children or adolescent or student).mp. and (children or adolescent or student).mp. and (Obeservational study or Cross-sectional or Case-control or Cohort or Prospective or Longitudinal).mp.	1726
	Scopus	(TITLE-ABS-KEY (Educational level OR Maternal education OR Parental education OR Effect of education OR Parental characteristics OR Socioeconomic status OR Social determinant) AND TITLE-ABS-KEY (Body OR Height OR Body weight OR Overweight OR Obesity OR Body mass index) AND TITLE-ABS-KEY (Children OR Adolescent OR student)) AND TITLE-ABS-KEY (Obeservational study or Cross-sectional or Case-control or Cohort or Prospective or Longitudinal)	1976
	CNKI	(Title/Keyword/Abstract=education status + parental education level + Socioeconomic status) AND (Title/Keyword/Abstract=body weight + height + obesity + overweight + body mass intake) AND (Keyword =children + adolescent + student) AND (Text Word = observational + cross-sectional + case-control + cohort +prospective)	568

Supplemental table 2. Search strategy to find potential relevant articles for inclusion in the meta-analysis

Topic	Database	Search terms	Retrieval
Nutrition policy and risk of obesity and/or overweight	PubMed	((Nutrition program [Title/Abstract]) OR (Nutrition policy [Title/Abstract]) OR (Nutrition intervention [Title/Abstract]) OR (sugar-sweetened beverage tax) OR (soda tax))AND ((Body height [MeSH Terms]) OR (Body weight [MeSH Terms]) OR (Overweight [MeSH Terms]) OR (Obesity [MeSH Terms]) OR (Body Mass Index [MeSH Terms])) AND ((children [Title/Abstract]) OR (adolescent [Title/Abstract]) OR (student [Title/Abstract])) AND ((Obeservational study [Text Word]) OR (Cross-sectional [Text Word]) OR (Case-control [Text Word]) OR (Cohort [Text Word]) OR (Prospective [Text Word]) OR (Longitudinal [Text Word]))	1951
	Web of Science	((AB=(Nutrition program) OR AB=(Nutrition policy) OR AB=(Nutrition improvement measures) OR AB=(sugar reduction program) OR AB=(sugar sweetened beverage tax) OR AB=(soda tax) OR OR (AB= (Nutrition Standards) OR (AB= (nutrition recommendations) OR AB=(wellness policies)) AND AB=((Body height) OR (Body weight) OR (Overweight) OR (Obesity) OR (Body Mass Index)) AND ((AB=(children) OR AB=(adolescent) OR AB=(student)) AND (AB=(Obeservational study) OR AB=(Cross-sectional) OR AB=(Case-control) OR AB=(Cohort) OR AB=(Prospective) OR AB=(Longitudinal))	2294
	Embase	((Nutrition program or Nutrition policy or Nutrition improvement measures or sugar reduction program or sugar-sweetened beverage tax policy or soda tax). ab. and (Body height or Body weight or Overweight or Obesity or Body Mass Index)). ab. and (Children or Child or Students). ti. and (children or adolescent or student).mp. and (Obeservational study or Cross-sectional or Case-control or Cohort or Prospective or Longitudinal).mp.	147
	Scopus	(TITLE-ABS-KEY (Nutrition program OR Nutrition policy OR Nutrition improvement measures OR sugar-sweetened beverage tax OR soda tax) AND TITLE-ABS-KEY (Body OR Height OR Body weight OR Overweight OR Obesity OR Body mass index) AND TITLE-ABS-KEY (Children OR Adolescent OR student)) AND TITLE-ABS-KEY (Obeservational study or Cross-sectional or Case-control or Cohort or Prospective or Longitudinal)	176
	CNKI	(Title/Keyword/Abstract= nutrition program + nutrition policy + nutrition improvement measures) AND (Title/Keyword/Abstract=body weight + height + obesity + overweight + body mass intake) AND (Keyword =children + adolescent + student) AND (Text Word = observational + cross-sectional + case-control + cohort +prospective)	216

Supplemental table 3. General characteristics of included studies

**First author (year),**<br>**Country, study name (study year)**	**Population**	**Follow-up (years)**	**Study type, quality score** [1]	**Exposure and assessment method**	**Outcome and assessment method**	**Covariates**	**Findings**
Bel-Serrat (2019) [1], Ireland, The WHO European Childhood Obesity Surveillance Initiative (COSI) (2008, 2015)	2755 (girls: 53.7%) children aged 6-10 years old at baseline	3	Cohort	Fresh fruit and vegetable intake at baseline (group 1: every day/most day; group 2: Sometimes/never)	Overweight and obesity at follow, up according to the International Obesity Task Force (IOTF) cut, offs	Adjust for measurement round, time follow, up, age, sex, baseline z, BMI, baseline abdominal obesity status, school socioeconomic status, school location, and household ownership (rented vs. owned)	Prevalence of overweight and obesity at follow, up:
			7	Parental completion questionnaires			Fresh fruit intake at baseline
							Sometimes/never: OR=1.88 (1.13, 3.12)
							Every day/most day: ref
							Vegetables intake at baseline
							Sometimes/never: OR=0.97 (0.59, 1.59)
							Every day/most day: ref
Chang (2012) [2], China, Shenyang children study (2010)	6777 (girls: 47.7%) children aged 6-18 years old	0	Cross-sectional	Enjoy eating vegetable or not	Obesity was defined by BMI with the criteria of the Working Group on Obesity in China (WGOC)	Adjust for maternal age, maternal obesity, paternal obesity, overeating, good appetite, too little physical activity, preference for meat, home computer, maternal age, preference for dairy	vegetable Yes: OR=0.796 (0.731, 0.867)
			8	Enjoy consuming sweetened beverage or not			SSB Yes: OR= 1.290 (1.174, 1.418)
				Self-completion questionnaires			No: ref

Chen (2007) [3], China, Guangzhou children study (2005)	752 (girls: 47.9%) children aged 3-7 years old	0	Cross-sectional	Enjoy eating vegetable or not	Obesity was defined by BMI with the criteria of a survey study on the physical development of children under 7 years old in 9 cities in China in 1995	Adjust for appetite, snack, being able to eat at kindergarten, dessert	Yes: OR= 0.67 (0.47, 0.95)
			8	Parental completion questionnaires			No: ref
Chen (2017) [4], China, Shenzhen children study (2015)	5086 (girls: 49.0%) children aged 9-15 years old	0	Cross-sectional	Eating vegetables yesterday or not	Obesity was defined by BMI with the criteria of the Working Group on Obesity in China (WGOC)	Adjusted for Fast eating, preference for sweet foods, preference for Western fast food, preference for sugary drinks, parental intervention in the amount of food the child eats, parental control of the child's diet structure, parental concern for the child's weight	Yes: OR= 0.628 (0.437, 0.912)
			8	Self-completion questionnaires			No: ref
Chen (2021) [5], China, Jiangxi children study (2020)	77,780 (girls: 45.9%) children aged 6-18 years old	0	Cross-sectional	Consumption frequency of fresh fruits (group 1: <1 times/d; group 2: >=1 times/d)	Overweight/obesity was defined by using age, and sex, specific BMI cutoff points according to the growth standards of China "Screening for overweight and obesity among school, age children and adolescents (WS/T 586, 2018)	Adjusted for frequency of eating breakfast, other diets, economic zone, monitoring site, school section, gender, sports	Consumption frequency of fresh fruits (>=1 times/d)
			8	Self-completion questionnaires			Overweight: OR=1.15 (1.10, 1.20)
							Obesity: OR=1.18 (1.11, 1.26)
							Consumption frequency of vegetables (>=1 times/d)
							Overweight: OR=1.12 (1.06, 1.19)
							<1 times/d: ref

Dudas (2008) [6], USA, Presenting for care to an urban hospital pediatric clinic and urgent care centerin Baltimore, Maryland (2006)	100 (girls: 42%) children aged 8-18 years old	0	Cross-sectional	Consume fruit or vegetable every day	Overweight was defined by the 2000 Centers for Disease Control growth charts	Adjust for age, gender, race, income, and bike ownership	Eat vegetable every day
			8	Consume soda every day or not			No: OR=3.1 (1.1, 8.6)
				20-item FFQ			Yes: ref
							Eat fruit weekly or less
							No: OR=1.8 (0.5, 6.0)
							Yes: ref
							SSB every day or not:
							No: OR=0.8 (0.3, 2.0)
							Yes: ref
Dupuy (2011) [7], France, The WHO, collaborative Health Behaviour in School, Aged Children (HBSC) survey (2009)	7154 (girls: 50.3%) children aged 11-15 years old	0	Cross-sectional	Consume fruit or vegetable every day	Overweight was defined by the International Obesity Task Force	Adjust for family affluence, smoking, consuming alcohol weekly, eating breakfast daily, PA, using video games, computer, interaction age*gender, interaction age*gender	Eating fruit daily
			8	Self-completion questionnaires			Yes OR=1.10 (0.90, 1.36)
							No: ref
							Eating vegetables daily
							Yes: OR=1.01 (0.84, 1.22)
							No: ref

Supplemental table 3. General characteristics of included studies

**First author (year),** **Country, study name (study year)**	**Population**	**Follow-up (years)**	**Study type, quality score** [1]	**Exposure and assessment method**	**Outcome and assessment method**	**Covariates**	**Findings**

Flores (2013) [8], USA, The Birth Cohort of the Early Childhood Longitudinal Study (ECLS, B) (2001)	6800 (girls: 55.7%) children aged 5-7 years old at baseline	7	Cohort	Ate fruit at least once in past 7 days at kindergarten age	Severe obesity (BMI >=99th percentile) was defined by stand unassisted on a SECA scale (Hamburg, Germany)	Adjust for BMI at 2 years old and preschool age, mother severely obese, drank tea or coffee, maternal gestational diabetes, latino, multiracial, BMI percentile at 9 months old, number of adults in househol, middle upper, arm circumference at 9 months old, maternal age at birth of first child, age, ever attended center, based child care, birth weight	Fruit:
			9	Drank sugary beverage at kindergarten age at least once in the past 7 days			Yes OR=0.3 (0.1, 0.7)
				Personal interviews			No: ref
							Sugar:
							Yes OR= 2.3 (1.4-3.7)
							No: ref
Guan (2018) [9], China, Foshan children study (2015)	1561 (girls: 45.53%) children aged 12-22 years old	0	Cross-sectional	Consumption frequency of fresh vegetable (group 1: =<1 times/d; group 2: 1-6 times/week; group 3: 0 times/week)	Obesity/overweight was defined by BMI with the criteria of the China Obesity Task Force in 2004	Adjusted for school type, gender, per capita household income, residence, sports, diet	Vegetable: 1, 6 times/week: OR=0.066 (0.023, 0.195)
			8	Drank sugary drinks: frequently, often or			0 times/week: OR=0.035 (0.012, 0.103)

				never			
				Self-completion questionnaires			=<1 times/d: ref
							Drank sugary drinks:
							Frequently: OR=5.477 (2.168, 13.839)
							Often: OR=2.666 (1.128, 6.304)
							Never: ref
Guo (2011) [10], China, Wuhan children study (2010)	1112 (girls: 48.7%) children aged 5.0±0.9 years old	0	Cross-sectional	Eating vegetables and fruits frequently	Obesity/overweight was defined by BMI with the World Health Organization and the International Obesity Task Force.	Adjusted for sex, age, birth weight, whether exclusively breastfed for 4 months, child's dietary habits	Eating vegetables and fruits frequently
			8	Parental completion questionnaires			Yes: OR=0.659 (0.477, 0.911)
							No: ref
Tang (2010) [11], Vietnam, Conducted in Ho Chi Minh City (2004)	2660 (girls: 50.0%) children aged 11-16 years old	0	Cross-sectional	Vegetables/fruit consumed: Both frequently;	Overweight and obesity was defined by the International Obesity Task Force	Adjust for gender, age, pubertal stage, residence, school location, household, parental characteristics, dietary intake, PA, energy expenditure	Vegetables/fruit:
			8	Vegetables frequently;			Both frequently: OR=0.3 (0.2, 0.4)
				Soft drink consumed: Frequently, not frequently, Never			Vegetables frequently: OR=0.5 (0.3, 0.7)
				Fruit frequently; Neither frequently			Fruit frequently: OR=0.4 (0.3, 0.6)
				FFQ			Neither frequently: ref
							Soft drink:
							Frequently: OR=3.0 (1.6, 5.3)
							Not frequently: OR=1.4 (1.1, 1.9)
							Never: ref

Huang (2017) [12], China, Luzhou children study (2013, 2016)	3000 (girls: 48.33%) children aged 6-12 years old	0	Cross-sectional	Enjoy eating vegetable or not	Obesity was defined by BMI with the World Health Organization	Adjusted for forcing the child to eat a certain food, asking the child to eat more, eating fast	Yes: OR= 0.632 (0.431, 0.874)
			8	Self-completion questionnaires			No: ref
Jia (2013) [13], China, Wenzhou children study (2010)	758 children aged 6-13 years old	0	Case-control	Frequency of vegetable intake	Obesity was defined by BMI with the criteria of the Working Group on Obesity in China (WGOC)	Adjusted for age, gender, frequency of other food intake, travel patterns, exercise time, housework time, sedentary time, sleep time	Frequency of vegetable intake
			9	Frequency of fruit intake			OR=0.765 (0.602, 0.976)
				Self-completion questionnaires			Frequency of fruit intake
							OR=1.185 (0.929, 1.513)
Kolmaga (2019) [14], Poland, conducted in Lodz (2008-2012)	622 (girls: 49.7%) children aged 12-18 years old	0	Cross-sectional	Everyday consumption of fruit and vegetables	Overweight and obesity were interpreted on the basis of centile charts of Lodz children	Adjust for gender, age, food behavior, food intake, hours of use of television, computer, physical activity, mother's education, encouragement, sleep	<1 time/d: OR=1.86 (1.01, 3.44)
			8	Self-completion questionnaires			>=1 time/d: ref

Supplemental table 3. General characteristics of included studies

**First author (year), Country, study name (study year)**	**Population**	**Follow-up (years)**	**Study type, quality score [1]**	**Exposure and assessment method**	**Outcome and assessment method**	**Covariates**	**Findings**
Li (2021) [15], China, conducted in Fujian province (2020)	28,006 (girls: 45.8%) children aged 3-7 years old	0	Cross-sectional	Everyday consumption of fruit and vegetables	Overweight and obesity were defined by BMI with the World Health Organization	Adjusted for the current appetite, eating speed, meat small food, puffed food, milk, often eat foreign fast food,	fruit and vegetables:
			8	Drink sugary beverages daily or not			Overweight group

				Parental completion questionnaires		restaurant meal frequency	Yes: OR=0.973 (0.890, 1.064)
							No: ref
							Obesity group
							Yes: OR=0.880 (0.789, 0.981)
							No: ref
							sugary beverages:
							Overweight group
							Yes: OR= 1.072 (0.69, 1.186)
							No: ref
							Obesity group
							Yes: OR= 1.179 (1.044, 1.332)
							No: ref
Lu (2018) [16], China, conducted in Beijing (2015)	1260 (girls: 46.51%) children aged 9-12 years old	0	Cross-sectional	Everyday consumption of fruit and vegetables	Obesity was defined by BMI with criteria for school, age children and adolescents in China	Adjusted for Eating Western fast food >3d in the past 7 days, eating at a faster rate, active at least 1h per day <3d, participating in extracurricular physical activity <3d, eating breakfast <5d in the past 7 days, eating Western fast food >3d in the past 7 days, eating before bed >3d in the past 7 days, eating at a faster rate, moderate intensity physical activity <3d, participating in extracurricular physical activity <3d, sedentary	In the past 7 day
			8	Self-completion questionnaires			Fruit:
							Yes: OR=1.29 (0.91, 1.82)
							No: ref
							Vegetable:
							Yes: OR=0.99 (0.68, 1.44)
							No: ref

						behavior	
Luo (2020) [17], China, Anyang area of Henan Province	548 (girls: 43.1%) children aged 5-12 years old	0	Cross-sectional	Dark vegetables for dinner	Overweight/obesity was defined by using age, and sex, specific BMI cutoff points according to the growth standards of China “Screening for overweight and obesity among school, age children and adolescents (WS/T 586, 2018)	Adjusted for other dietary status, physical activity, number of night-wakings per day	Yes: OR=0.73 (0.46, 0.92)
-2019			8	Personal interviews			No: ref
Nie (2014) [18], China, Qinghai children study (2010)	2597 (girls: 47.6%) children aged 3-6 years old	0	Cross-sectional	Enjoy eating vegetable or not	Simple obesity was defined by BMI with the World Health Organization	Adjusted for age, supplementation time, genetic factors, birth weight, appetite, amount of food per meal, picky eaters, TV time, eating speed, whether to eat fast food and fried food, exercise time, family eating habits	Yes: OR=1.500 (1.01, 2.22)
			8	Parental completion questionnaires			No: ref
Pengpid (2016) [19], Southeast Asian Nations	30,284 (girls: 51.5%) children aged 13-15 years old	0	Cross-sectional	Fruits consumption:	Overweight or obese was defined for international survey	Adjust for age, country income, dietary behavior and substance use, PA, physical factor, social familial factor.	Boys
(ASEAN), The Global School, based Student Health Survey (GSHS) from seven ASEAN members			8	>=2 servings/d			Fruits

(2007-2013)				Vegetables:			>=2 servings/d: OR=1.16 (0.96, 1.39)
				>=3 servings/d			<2 servings/d: ref
				Self-completion questionnaires			Vegetables
							>=3 servings/d：OR=1.34 (1.09, 1.65)
							<3 servings/d: ref
							Girls
							Fruits
							>=2 servings/d: OR=1.02 (0.86, 1.20)
							<2 servings/d: ref
							Vegetables
							>=3 servings/d: OR=1.13 (0.89, 1.43)
							<3 servings/d: ref
Qin (2013) [20], China, conducted in Chongqing (2011)	3639 (girls: 50.3%) children aged 6-18 years old	0	Cross-sectional	Vegetable intake frequency	Obesity was defined by BMI with the criteria of the Working Group on Obesity in China (WGOC)	Adjusted for birth mass, high energy diet, family history of obesity, maternal smoking, breastfeeding, vitamin supplementation, exercise schedule, other dietary status	>=2 times/d: OR=0.714 (0.536, 0.950)
			8	Self-completion questionnaires			<2 times/d: ref

Supplemental table 3. General characteristics of included studies

**First author (year),**<br>**Country, study name (study year)**	**Population**	**Follow-up (years)**	**Study type, quality score [1]**	**Exposure and assessment method**	**Outcome and assessment method**	**Covariates**	**Findings**

Qiu (2020) [21], China, conducted in Henan (2018)	5146 (girls: 49.2%) children aged 12.12±1.14 years old	0	Cross-sectional	Everyday consume vegetables	Obesity was defined by using age and sex, specific BMI cutoff points according to the growth standards of China “Screening for overweight and obesity among school, age children and adolescents (WS/T 586, 2018)	Adjusted for Speed of eating, preference for desserts, western food, control and intervention of children's dietary structure and intake	Yes: OR=0.629 (0.439, 0.925)
			8	Self-completion questionnaires			No: ref
Salahuddin (2017) [22], USA (Low, Income, Predominantly Hispanic/Latino), The Texas Childhood Obesity Research Demonstration (TX CORD) Study (2012, 2014)	517 (girls: 50.9%) children aged 2-12 years old	0	Cross-sectional	Child’s fruit and vegetable consumption	The Centers for Disease Control and Prevention’s 2000 growth charts (severe obesity: BMI that was >=120% above the 95th percentile of sex, specific and age, specific BMI)	Adjust for child’s sex, child’s race/ethnicity, poverty income ratio <125% or not, parental marital status, parent’s physical activity (>=30 minutes per day of physical activity for >=5 days/week).	Age Group 2–5 y: OR=0.89 (0.70, 1.13)
			8	Number of times child ate fruits and vegetables yesterday			Age Group 6–8 y: OR=1.11 (0.91, 1.35)
				Questionnaire			Age Group 9–12y: OR=0.98 (0.81, 1.19)
							0 times/d=ref
Santiago (2013) [23], Spain, the program: Feed their health (2008)	2814 (girls: 49.6%) children aged 6-12 years old	0	Cross-sectional	Fruits consumption	Overweight or obesity was defined for the International Obesity Task Force (IOTF)	Adjust for extracurricular sports activities, breakfast every day, buns consumption, fast food consumption, sweets	Boys
			8	Group 1: <2/day: 66.1			>=2 times/day: OR=0.8 (0.6, 0.9)
				Group 2:>=2/day:33.9			<2 times/day: ref

				FFQ		consumption	
Wang (2016) [24], China, Five cities children study (2015)	3896 (girls: 46.7%) children aged 8-13 years old	0	Cross-sectional	Enjoy eating vegetables and fruits	Obesity was defined by using age, and sex, specific BMI cutoff points according to the growth standards of China "Screening for overweight and obesity among school, age children and adolescents (WS/T 586, 2018)	Adjusted for gender, city, age, eating behavior, snacking while online, parents and teachers encouraging healthy food, parents and teachers encouraging physical activity, physical activity	Yes: OR=0.753 (0.588, 0.963)
			8	Self-completion questionnaires			No: ref
Wei (2009) [25], China, conducted in Shenzhen (2005)	3836 (girls: 44.0%) children aged 7-10 years old	0	Cross-sectional	Often eating vegetables or fruits	Obesity and overweight were defined by BMI with the criteria of the Working Group on Obesity in China (WGOC)	Adjusted for gender, whether they like to eat fatty meat, whether they are always picky eaters, whether they often do outdoor exercise	vegetables or fruits
			8	Enjoy consuming soft drinking or not			Often: OR=0.486 (0.249, 0.951)
				Self-completion questionnaires			Not often: ref
							SSB
							Yes: OR=2.815 (1.009-7.847)
							No: ref
Wu (2019) [26], China, shanghai children study (2015)	2928 (girls: 51.98%) children aged 10-15 years old	0	Cross-sectional	Vegetables consumption	Obesity and overweight were defined by BMI with the criteria of the Working Group on Obesity in China (WGOC)	Adjusted for Age, gender, exercise within 1 week	<1 times/d: ref
			8	<1 times/d			1 times/d: OR=0.753 (0.598, 0.949)
				1 times/d			>1 times/d: OR=0.731 (0.585, 0.914)
				>1 times/d			
				Self-completion questionnaires			

Xiang (2011) [27], China, conducted in Inner Xining (2010)	1842 (girls: 46.9 %) children aged 3-6 years old	0	Cross-sectional	Enjoy eating vegetables and fruits	Simple obesity was defined by BMI with the World Health Organization	Adjusted for Age, supplementation time, father's body mass index, mother's body mass index, appetite, amount of food per meal, picky eaters, TV time, eating speed, whether to eat fast food and fried food often	Yes: OR=1.50 (1.01, 2.22)
			8	Parental completion questionnaires			No: ref
Yang (2021) [28], China, conducted in Inner Mongolia (2020)	186,649 (girls: 49.6 %) children aged 6-18 years old	0	Cross-sectional	Times of eating fruit/vegetable per day	Obesity was defined by using age, and sex, specific BMI cutoff points according to the growth standards of China "Screening for overweight and obesity among school, age children and adolescents (WS/T 586, 2018)	Adjusted for area, monitoring point, gender, school section, eating sweets, fried food, breakfast, exercise, number of physical education classes, TV time, sleep time, psychological condition	Times of eating fruit per day
			8	Self-completion questionnaires			<2 time/d: ref
							>=2 time/d: OR=1.07 (1.04, 1.11)
							Times of eating vegetable per day
							<2: ref
							>=2: OR=1.06 (1.03, 1.10)

Supplemental table 3. General characteristics of included studies

**First author (year),** **Country, study name (study year)**	**Population**	**Follow-up (years)**	**Study type, quality score [1]**	**Exposure and assessment method**	**Outcome and assessment method**	**Covariates**	**Findings**

Zeng (2010) [29], China, Yangchun primary school student study (2009)	1219 (girls: 44.5 %) children aged 6-12 years old	0	Cross-sectional	Enjoy eating vegetables	Obesity was defined by BMI with the criteria of the Working Group on Obesity in China (WGOC)	Adjusted for family situation, parents' situation, students' eating habits, living habits	Yes: OR=0.508 (0.368, 0.702)
			8	Self-completion questionnaires			No: ref
Zhang (2011) [30], China, conducted in Shanghai (2005)	12,337 (girls: 47.4 %) children aged 3-6 years old	0	Cross-sectional	Enjoy eating vegetables	Simple obesity was defined by BMI with the World Health Organization	Adjusted for child caregiver/parent, dominant, eating speed/fast, parental perception of child's size, mother's perception of child's exercise, mother's perception of child's food intake, whether grandparents restrict child's snacking	No: OR=3.854 (1.73, 8.57)
			8	Parental completion questionnaires			Yes: ref
Zhang (2013)-1 [31], China, conducted in Shandong (2011)	1584 (girls: 50.1 %) children aged 6-12 years old	0	Cross-sectional	Having fruit every day or not	Overweight and obesity were defined by BMI with the criteria of the Working Group on Obesity in China (WGOC)	Adjusted for Gender, birth weight, weight at week, father's BMI, mother's BMI, average monthly household income, daily sleep time, daily computer/TV time, whether or not the birth was normal, eating habits	<1 times/d: OR=1.381 (1.044, 1.826)
			8	Self-completion questionnaires			>=1 times/d: ref
Zhang (2013)-2 [32], China, conducted in Chongqing (2010)	2970 (girls: 47.7 %) children aged 6-12 years old	0	Cross-sectional	Enjoy eating fruit	Obesity was defined by BMI with the criteria of the Working Group on Obesity in China	Adjusted for Family feeding style, children's eating habits	Not: OR=1.79 (1.24, 2.60)
			8	Self-completion			Yes: ref

				questionnaires	(WGOC)		
Zou (2020) [33], China, conducted in Wuhan (2020)	7504 (girls: 45.3 %) children aged 12-18 years old	0	Cross-sectional	Having fruit and vegetable every day or not	Obesity was defined by using age, and sex, specific BMI cutoff points according to the growth standards of China "Screening for overweight and obesity among school, age children and adolescents (WS/T 586, 2018)	Adjusted for gender, school level, sleep deprivation, length of sleep, sleep disorder, mild daytime dysfunction, self, perceived good sleep quality, other dietary status, home, cooked breakfast, physical activity, static activity	Not: OR=0.951 (0.861, 1.049)
			8	Self-completion questionnaires			Yes: ref
Zurriaga (2011) [34], Spian, The OBICE (OBesidad Infantil en redes CEntinelas) study	1188 (girls: 44.4 %) children aged 3-14 years old	0	Case-control	Consuming fruit for dessert	Obesity was defined by 'Fundacio´n Orbegozo, Sobradillo' tables	Adjust for parental obesity, siblings' obesity, regular consumption of breakfast, five meals per day, eating between meals, consumption of meat	fruit for dessert
(2007, 2008)			9	Sweets and soft drinks consumption			Yes: OR=0.6 (0.4, 0.8)
				Questionnaire			No: ref
							SSB:
							Not available: OR=2.1 (0.8, 5.4)
							>2 times/week: OR=2.0 (1.4, 2.9)
							<=2 times/week: ref
Beck (2014) [35], US, the San Francisco Bay Area study (2010)	319 children aged 8-10 years	0	cross-sectional	Soda intake: additional serving of 240 ml	Overweight and Obesity classified as US National Center for Health Statistics	Adjusted for age, gender, the retained beverage variables	Yes: 1.29 (1.13, 1.47)

			7	Self-administrated questionnaire	growth charts		No: ref
Calvo (2014) [36], Spain, The GENOI (Grupo Navarro de Estudio de la Obesidad Infantil) case-control study (2001)	348 (girls: 47.1%) children aged 5.5-18.8 years old	0	Case-control	SSCB, Sugar-sweetened carbonated beverage (group 1: Never or almost never; group 2: <1 serving/week; group 3: 1–4 servings/week; group 4: >4 servings/week)	Obesity was defined according to the Spanish BMI reference charts	Adjust for sex, age, total energy intake, physical activity, sedentary behavior (time spent watching television or using a computer), fast-food consumption (pizza, hamburgers and sausages) and other sugar-sweetened beverage consumption (fruit-flavored juices)	Never or almost never: ref
	174 cases and 174 matched controls		9	Semi-quantitative FFQ			<1 serving/week: OR=1.11 (0.46, 2.69)
							1–4 servings/week: OR=1.52 (0.75, 3.08)
							>4 servings/week: OR=3.46 (1.24, 9.62)
Cao (2008) [37], China, conducted in Urumqi, Xinjiang province (2005)	4063 (girls: 48.1%) children aged 3-7 years old	0	Cross-sectional	Sweetened beverages account for more than 1/4 of daily water intake	Obesity was defined by BMI with the World Health Organization	Adjusted for appetite, high food intake, gender, meat, complementary food addition	Yes: OR=4.513 (2.763, 7.648)
			8	Parental completion questionnaires			No: ref
de Bont (2021) [38], Spain, ECHOCAT project (2017-2019)	2213 children aged 9–12 years	0	cross-sectional	Sugar-sweetened beverage:> 1 times/day	Overweight and Obesity was defined by the World Health Organization Growth Reference	Adjusted for study design, sex, age, maternal education and area-level SES.	Yes: 1.14 (0.94, 1.37)
			8	FFQ			No: ref

Deng (2013) [39], China, conducted in Guangdong province (2010)	815 (girls: 45.8%) children aged 3-5 years old	0	Cross-sectional	Enjoy consuming sweet drinks or not	Obesity was defined by BMI with the World Health Organization	Adjusted for at least one parent obesity, eating before bedtime, good family conditions, activity, knowledge, food consumption, sleep time >10 h, frequent consumption of Western-style fast food, frequent snacking, awareness of the dangers of obesity and prevention	Yes: OR=2.17 (1.76, 3.16)
			8	Parental completion questionnaires			No: ref
Ding (2019) [40], China, Nanjing children study (2015)	311 (girls: 42.4%) children aged 3-6 years old	0	Cross-sectional	Consume sweet drinks every day or not	Obesity was defined by BMI with the World Health Organization	Adjusted for caregiver's education level, family income, number of meals per day, whether daily intake of coarse grains, whether daily consumption of fried food, whether weekend exercise	Yes: OR=2.609 (1.139, 6.206)
			7	Parental completion questionnaires			No: ref

Supplemental table 3. General characteristics of included studies

**First author (year),**<br>**Country, study name (study year)**	**Population**	**Follow-up (years)**	**Study type, quality score** [1]	**Exposure and assessment method**	**Outcome and assessment method**	**Covariates**	**Findings**
Gui (2017) [41], China, A national multicenter intervention program against	53151 (girls: 49.8%) children aged 6-17 years old	0	Cross-sectional	SSB Frequency/week: High Frequency, Medium Frequency, None Frequency	Overweight and obesity was defined by BMI with the criteria of the Working Group on	Adjust for age, sex, residence, maternal education, paternal education, family income, screen time,	Overweight:

obesity in Chinese children and adolescents (2013)			8	A self-reported questionnaire for children	Obesity in China (WGOC)	physical activity, meat and fried food	High Frequency OR=1.063 (0.985, 1.148)
							Medium Frequency OR=1.033 (0.952, 1.120)
							None Frequency ref
							Obesity:
							High Frequency: OR=0.992 (0.915, 1.076)
							Medium Frequency: OR=0.995 (0.913, 1.084)
							None Frequency: ref
Haboush-Deloye (2021) [42], US, the KHS survey (2012)	7814 children aged 4-6 years	0	cross-sectional	Soda intake: any weekly consumption	Overweight and Obesity was defined by the CDC criteria	Adjusted for SES, gender, PA, screen time, feeding practice at 6 months	Yes: 1.21 (1.07, 1.36)
			6	7-days record			No: ref
Hatami (2014) [43], Iran, the Tehran study (2009-2010)	1109 children aged 10-18 years	0	cross-sectional	Sugar-sweetened beverage: 5-7 times/week	Overweight and Obesity was defined by the World Health Organization Growth Reference	Adjusted for age, sex	Yes: 1.35 (0.98, 1.88)
			7	FFQ			No: ref
Heo (2020) [44], US, the Centers for Disease Control and Prevention (CDC) Youth Risk Behavior Surveillance data (2013)	13,571 children aged 10-18 years	0	cross-sectional	Soda intake: 2 times/day	Obesity was defined following the CDC criteria	Adjusted for age, Hispanic ethnicity	Yes: 1.34 (1.17, 1.55)
			7	Self-administrated questionnaire			No: ref

Hwang (2020) [45], Korea, KNHANES (2018)	6121 children aged 10-18 years	0	cross-sectional	Sugar-sweetened beverage: > median consumption (boy: 280.5g, girl: 210g)	Overweight and Obesity was defined by the 2017 Korean National Growth charts	Adjusted for age, sex, BMI, household income level, residential area, energy intake	Yes: 1.10 (0.83, 1.47)
			7	24h dietary recall			No: ref
Karki (2019) [46], Nepal, Conducted in May–October (2017)	575 (girls: 43%) children aged 6-13 years old	0	Cross-sectional	Soft drinks: more than a bottle (> 250 ml)/week; Less than a bottle/week or never drink	Overweight and obesity was defined by BMI with the World Health Organization	Adjust for age, sex, education level of parents, occupation, monthly income, birth weight, family type, ethnicity, dietary characteristics, physical activity level, mode of transport to school, sedentary behavior	Yes: OR=1.13 (0.63, 2.05)
			8	The SPANS 2010 questionnaire for assessing the last 7 days			No: ref
Leon-Guerrero (2020) [47], US, the CHL program (2013)	634 children aged 2-8 years	0	cross-sectional	Sugar-sweetened beverage:>1.09 cups/day	Obesity was defined following the CDC criteria	Adjusted for community, age, sex, ethnicity	Yes: 1.81 (1.11, 3.01)
			7	2 day dietary recalled			No: ref
Lim (2009) [48], USA, the Detroit Dental Health Project (2002-2005)	365 children aged 3-7 years	2	Cohort	Beverage intake (oz/day)	overweight was defined as 85th percentile ≤ BMI <95th percentile, obese was defined as BMI ≥95th percentile.	Adjusted for age, gender, caregiver's education and income, and child's baseline dietary energy intake	Yes: 1.04 (1.01, 1.07)
			8	FFQ			No: ref
Liu (2012) [49], US, NHANES (2006)	7854 children aged 12-19 years	0	cross-sectional	Sugar-sweetened beverage: >24 oz./day	Overweight and Obesity was defined by the CDC criteria	Adjusted for Age, race/ethnicity, perceived health, household income level, reference person's education, region, survey year, total energy intake	Yes: 1.18 (0.95, 1.47)
			8	24h dietary recall			No: ref

Li (2016)-1 [50], China, Xi'an children study (2013)	1466 children aged 3-6 years old	0	Case-control	Daily consumption of sweetened beverage	Overweight and obesity was defined by BMI with the World Health Organization	Adjusted for parental obesity, high family income, frequent consumption of sweet drinks, good appetite, fast eating, high parental education, surrogate caregivers for parents	Yes: OR=1.351 (0.958, 1.904)
	Case group: 701 children		9	Parental completion questionnaires			No: ref
	Control group: 765 children			Parental completion questionnaires			
Li (2016)-2 [51], China, conducted in Shanxi province (2013)	10,374 (girls: 47%) children aged 0-7 years old	0	Cross-sectional	Having sweet drink every day or not	Overweight and obesity was defined by BMI with the World Health Organization	Adjusted for appetite, speed of eating, preference for meat	Yes: OR=1.342 (1.005, 1.791)
			8	Personal face-to-face interview			No: ref
Li (2017) [52], China, conducted in Shanxi province (2013)	10,374 (girls: 47%) children aged 0-7 years old	0	Cross-sectional	Having sweet drink every day or not	Overweight and obesity was defined by BMI with the World Health Organization	Adjusted for parental education, whether the primary surrogate was a parent, birth weight, parental overweight and obesity, family income, appetite, meal speed, whether the average cumulative daily activity time was >120 min/d, whether the daily TV/computer time was <60 min/d, whether the child was exclusively breastfed within 6 months	Yes OR=1.351 (0.958, 1.904)
			8	Personal face-to-face interview			No ref

Liu (2017) [53], China, conducted in Chengde (2015)	16,811 (girls: 47.7%) children aged 6-17 years old	0	Cross-sectional	Like to drink sugary drinks	Overweight and obesity was defined by BMI with the criteria of the Working Group on Obesity in China (WGOC)	Adjusted for children younger than gestational age or older than gestational age, artificial feeding, parental (maternal) obesity and negativity, low self-esteem, binge drinking, eating fried foods and puffed foods, proportion of breakfast skipping, daily physical activity time less than 30 min, time spent watching TV online more than 2 h, insufficient sleep time	Yes: OR=2.5 (1.48, 5.36)
			8	Self-completion questionnaires			No: ref
Liu (2020) [54], China, conducted in Tianjin (2018)	5154 (girls: 49.9%) children aged 8-18 years old	0	Cross-sectional	Intake of sugary drinks/ one week: never drink; less than once a day; more than once a day	Overweight and obesity was defined by using age, and sex, specific BMI cutoff points according to the growth standards of China "Screening for overweight and obesity among school, age children and adolescents (WS/T 586-2018)	Adjusted for region, gender, school level, sleep time per day in a week, sweet food intake, and number of days with more than 60 min of moderate-to-vigorous exercise	More than once a day: OR=1.614(1.224-2.127)
			8	Self-completion questionnaires			Less than once a day: OR=1.276 (1.068-1.526)
							Never drink: ref
Martinez-Ospina (2019) [55], US, the Muevete Escolar	714 children aged 7-14 years	0	cross-sectional	Sugar-sweetened beverage:>1.09 cups/day	Overweight and Obesity was defined by the World Health	Adjusted for age, sex, socioeconomic status	Yes: 0.53 (0.28, 1.01)

school-based program (2015)			8	FFQ	Organization Growth Reference		No: ref
Nasreddine (2014) [56], Lebanon, the UNDP study (2009)	868 children aged 6-19 years	0	cross-sectional	SSB: High consumption (3rd tertile based on percent contribution to daily energy intake)	Overweight and Obesity was defined by the World Health Organization Growth Reference	Adjusted for baseline socio-demographic, lifestyle, dietary characteristics	Yes: 1.69 (0.76, 3.76)
			7	24-h recall			No: ref
Ochoa (2007) [57], Spain, Navarra region of Spain and control subject (2004)	185 children aged 6-18 years	0	case-control	Sugar-sweetened beverage: 1 additional serving/day	Obesity was defined by Spanish BMI reference data for age and gende	Adjusted for family obesity, physical activity, energy intake and watching	Yes: 1.74 (1.05, 2.89)
			7	FFQ			No: ref
Papandreou (2010) [58], Greek, the Thessaloniki student study (2007)	410 children aged	0	case-control	Sugar-sweetened beverages: 1 serving/day	Overweight and obesity based on IOTF criteria	Adjusted for energy intake	Yes: 1.92 (1.21, 2.78)
			7				No: ref
Payab (2015) [59], Iran, the CASPIAN-IV Study (2011-2012)	13,486 children aged 6-18 years	0	cross-sectional	Consum sugar-sweetened beverage or not	Body mass index (BMI) ≥ 95th percentile was considered as obesity.	Adjusted for family history of chronic disease, physical activity, screen time, socio- economic status	Yes: 1.18 (1.03, 1.35)
			7	Past 7 days			No: ref
Peng (2020) [60], China, conducted in Chengdu (2018)	1500 (girls: 46.3%) children aged 6-12 years old	0	Cross-sectional	Like consuming sugary drinks or not	Obesity is defined according to the diagnostic and efficacy assessment criteria of simple obesity disease	Adjusted for gender, huge baby, premature baby, parental obesity, late night snack, fast eating, meat-loving, fried food, dessert, insufficient exercise, parental perception of child size	Yes: OR=1.547 (1.089, 2.196)
			8	Self-completion questionnaires			No: ref

Pengpid (2016) [19], Southeast Asian Nations, The Global School-based Student Health Survey (GSHS) from seven ASEAN members	30,284 (girls: 51.5%) children aged 13-15 years old	0	Cross-sectional	One or more carbonated soft drinks per day	Overweight and obesity was defined by an international survey	Adjust for age, country income, dietary behavior, substance use, pa, physical factor, social family factor	Boys
(2007-2013)			8	Self-completion questionnaires			One or more times carbonated soft drinks per day: OR=0.99 (0.70-1.39)
							Girls
							One or more times carbonated soft drinks per day: OR=0.89(0.69-1.16)
							Didn't drink any soft drinks in the past 30 days: ref
Quah (2019) [61], Singapore, GUSTO study (2009)	767 children aged 5-7 years	1.5	Cohort	Sugar-sweetened beverage: High intake (198-328 ml/d) vs low intake (21-57 ml/d)	Overweight and Obesity was defined by the World Health Organization Growth Reference	Adjusted for ethnicity, education, birth weight for gestational age, screen time, breastfeeding duration and parity.	High intake: 1.01 (0.67, 1.81)
			8	FFQ			Low intake: ref
Rong (2018) [62], China, conducted in Shanghai (2015)	932 (girls: 48.18%) children aged 3-6 years old	0	Cross-sectional	Consume sweetened beverage or not	Overweight and obesity was defined by BMI with the World Health Organization	Adjusted for gender, parental BMI, birth weight, eating well at every meal, time spent outside of school	Frequently: OR= 3.73 (1.80, 7.74)
			8	Parental completion questionnaires			Seldom/never: ref
Sakaki (2019) [63], USA, The	26,554 (girls: 54%) children	0	Cross-sectional	Orange juice (OJ) intake	Overweight and obesity was defined	Adjust for cohort, age, race, total energy intake	Boys

Growing Up Today Study (1996-2004)	aged 9-16 years old		8	A semi-quantitative FFQ during the past year	by the U.S. Centers for Disease Control and Prevention	excluding orange juice, moderate/physical activity and screen time	obese
							Never or <1 Glass Per Month: ref
							>1 Glass per Day: OR=0.83 (0.59, 1.15)
							Overweight/obese:
							Never or <1 Glass Per Month ref
							>1 Glass per Day OR=0.91 (0.73, 1.13)
							Girls
							obese:
							Never or <1 Glass Per Month: ref
							>1 Glass per Day: OR=1.46 (1.01, 2.11)
							Overweight/obese:
							Never or <1 Glass Per Month: ref
							>1 Glass per Day OR=1.24 (1.02, 1.62)
Shen (2014) [64], China, conducted in Shanghai, Wuxi, Kunshan (2011)	443 (girls: 46.5%) children aged 3-6 years old	0	Cross-sectional	Orange juice (OJ) intake	Overweight and obesity was defined by BMI with the criteria of the Working Group on Obesity in China (WGOC)	Adjusted for age and sex	>=3 times/week: OR=1.9 (0.94, 3.76)
			8	3d-24h dietary recall and FFQ			<3 times/week: OR=1.1 (0.63, 2.23)
							Never: ref
Song (2020) [65], China, conducted in Shangrao, Jiangxi (2018)	1167 (girls: 47.7%) children aged 13-18 years old	0	Cross-sectional	Carbonated beverages	Overweight and obesity was defined by using age, and sex, specific BMI	Adjusted for age	>=4 times/week: OR=1.81 (0.37, 8.97)
			8	Self-completion questionnaires			1-3 times/week: OR=1.28 (0.45, 3.63)

					cutoff points according to the growth standards of China “Screening for overweight and obesity among school, age children and adolescents (WS/T 586-2018)		<1 times/week: OR=1.02 (0.54, 1.92)
							Never: ref
Shan (2010) [66], China-Beijing, the BCSCP study (2007)	21,198 children aged 6-18 years	0	cross-sectional	Consum sugar-sweetened beverage or not	Overweight and Obesity was defined by the World Health Organization Growth Reference	Adjusted for age, gender, Tanner stage, urban/rural residence	Yes: 1.06 (0.92, 1.20)
			7	Self-administrated questionnaire			No: ref
Tan (2012) [67], China, Shandong children study (2010)	1584 (girls: 50.1%) children aged 6-12 years old	0	Cross-sectional	Weekly sugary drinks drinking frequency	Overweight and obesity was defined by BMI with the criteria of the Working Group on Obesity in China (WGOC)	Adjusted for average daily consumption of vegetables and fruits, average daily time spent watching TV and playing computer, average daily outdoor exercise time	Yes: OR=1.206 (1.048, 1.387)
			8	Self-completion questionnaires			No: ref
Valente (2011) [68], Portugal, the Porto study (2007)	1675 children aged 5-10 years	0	cross-sectional	SSB: >3 servings/day	Overweight and Obesity classified as recommended by the International Obesity Task Force	Adjusted for Energy intake, parents’ education level, time of sleep, questionnaire responder, total carbohydrates, sugars, MUFA, television watching	Yes: 0.63 (0.39, 1.04)
			7	FFQ			No: ref
Vinciguerra (2019) [69], Italy, the Catania	1702 children aged 6-15 years	0	cross-sectional	Consum sugar-sweetened beverage or not	Overweight and obesity based on IOTF criteria	Adjusted for gender, level of PA ST, SSB, parental risk factors	Yes: 0.92 (0.72, 1.13)

metropolitan area study (2014)			7	FFQ			No: ref
Wang (2018) [70], korea, KNHANES (2013)	1520 children aged 6-11 years	0	cross-sectional	Carbonated beverage intake of > 200 mL/day	Overweight and Obesity was defined by the 2017 Korean National Growth charts	Adjusted for age, sex, household income, region, energy density	Yes: 2.41 (1.35, 4.33)
			7	24-hour recall			No: ref
Wang (2021) [71], China, China Health and Nutrition Survey (1997-2011)	1916 (girls: 48.7%) children aged 7-17 years old	0	Cross-sectional	Times of intake of sugar-sweetened beverages	Central obesity was defined by waist circumference with a study of waist circumference cut-off points for school-age children and adolescents aged 7-18 years in China	Adjusted for age, gender, urban and rural areas, per capita household income, total energy intake, sleep	>=3 times/week: OR=1.680 (1.194-2.364)
			8	Self-completion questionnaires			1-2 times/week: OR=1.233 (0.901-1.688)
							1-2 times/month: OR=1.158 (0.837-1.601)
							<1 times/month: ref
Wu (2022) [72], China, Shanghai children study (2015)	897 (girls: 47.9%) children aged 3-6 years old	0	Cross-sectional	Times of carbonated drinks	Overweight and obesity was defined by BMI with the criteria of the Working Group on Obesity in China (WGOC)	Adjusted for gender, age, family income, daily time spent outdoors in the garden, parental overweight/obese	Yes: OR=2.92 (1.37-6.13)
			8	Parental completion questionnaires			No: ref
Yu (2023) [73], Jiangsu Province of China, Surveillance for Common Disease and Health Risk Factors Among Students in Jiangsu Province	119,467 students (girl: 47.5%) aged 8-17 years	0	Cross-sectional	Frequent drinking sugar drinks	Overweight/obesity was defined by a standard established by the China Obesity Task Force	Adjusted for age, gender, residence, regions of the province, family types, and paternal education and maternal education, and physical activity	>1 time/d: OR=1.05 (1.02, 1.07)
			8	Self-completion questionnaires			Never: ref

(2021–2022)							
Zhang (2019) [74], China, Jiangsu children study (2017)	32,055 (girls: 49.3%) children aged 6-18 years old	0	Cross-sectional	Consuming sugar-sweetened beverage	Overweight and obesity was defined by guidelines for the Prevention and Control of Overweight and Obesity in School-aged Children and Adolescents in China"	Adjusted for region, place of residence, school section, gender, milk drinking, high intensity exercise	>1 time/d: OR=1.18 (1.04, 1.33)
			8	Self-completion questionnaires			< 1time/d: OR=1.14 (1.06, 1.22)
							Never: ref
Zhang (2018) [75] , China, conducted in Beijing (2015)	1327 (girls: 49.6%) children aged 0-6 years old	0	Cross-sectional	Frequent drinking sugar drinks	Obesity was defined by BMI with the World Health Organization	Adjusted for age, parental BMI, whether medication was used during pregnancy, whether multiples were born, mode of delivery, average total daily sleep time, frequency of eating fast food, whether sleeping after 23:00 regularly, eating speed, number of complete meals per day	Never: OR=1.571 (0.428, 5.765)
			8	Parental completion questionnaires			Every day: ref
Andegiorgish (2012) [76], China, conducted in Tianjin (2010)	3140 (girls 50.4%) children aged 7-18 years	0	Cross-sectional	Mother's or Father's educational level:	Overweight was defined by age- and sex-specific BMI cut-off points with the Working Group for Obesity in China (2004)	Adjusted for paternal obesity and maternal obesity in the regression model for each independent factor	Mother:
			7	Primary or lower			Primary or lower: ref
				Secondary or junior			Secondary or junior: OR=1.66 (1.20–2.29)
				Tertiary or above			Tertiary or above: OR=2.97 (2.03–4.36)
				Questionnaire			Father:

							Primary or lower: ref
							Secondary or junior: OR=1.33 (0.92–1.93)
							Tertiary or above: OR=2.48 (1.63–3.78)
Androutsos (2018) [77], Belgium, Bulgaria, Germany, Greece, Poland and Spain, ToyBox-study (May/June 2012)	7541 (girls 48.0%) children aged	0	Cross-sectional	Maternal education:	Overweight and obesity were defined by BMI with the International Obesity Task Force (IOTF) cut-off points	Adjusted for age, gender, sociodemographic factors, perinatal factors, energy balance-related behaviors	Mother:
			8	Low (≤14 years)			>14 years: ref
				Mid/High (>14 years):			≤14 years: OR=1.23 (0.96-1.60)
				Questionnaire			Father:
							>14 years: ref
							≤14 years: OR=1.03 (0.81-1.32)
Bhuiyan (2013) [78], Bangladesh, conducted in Dhaka (2007)	198: 99 cases /overweight (girls 44%), 99 controls (girls 47%) children aged 10-15 years	0	Case-control	Maternal education	Overweight and obesity were defined by the age- and sex-specific growth chart of the US Centers for Disease Control and Prevention	n/a	Up to higher secondary: ref
			8	Up to higher secondary			Graduation degree or more: OR=1.1 (0.6-2.0)
				Graduation degree and more			
				Questionnaire			
Bibiloni (2010) [79], Spain, conducted in Balearic Islands (2007-2008)	1231 (girls 53.4%) children aged 12-17 years	0	Cross-sectional	Parental educational level: Low (< 6 years at school)	Obesity was defined by the WHO growth standards for children and adolescents	Adjusted for age, place of birth, parental SES status, hours of sleep, number of meals, breakfast habit, sweets or salty snacks, attention to mass media, alcohol consumption, current smoking habit, physical activity level	Boys:
			8	Medium (6 –12 years of education)			Low: OR=3.47 (1.58-7.62)
				High (>12 years of education Questionnaire)			Medium: OR=2.35 (1.04-5.34)
							High: ref
							Girls:

							Low: OR=3.29 (1.38-7.89)
							Medium: OR=1.89 (0.73-4.94)
							High: ref
Brophy (2009) [80], UK, Millennium Cohort Study	17561 children aged 5 years	0	Cross-sectional	Primary carer's education level:	Obesity was defined by the International Obesity Task Force (IOTF) age and sex specific BMI cut offs	Adjusted for ethnic group, Birth weight, Physical activity, Indoor activities, Time spent in watching television, Family income	No qualification: ref
			8	No qualifications			Leaves school after age 16: OR=0.63 (0.52-0.63)
				Education to age 16			
				Education to age 18			
				University (education to age 20+)			
				Questionnaire			
Chen (2012) [81], China Taiwan, Taiwan Children Health Study cohort (2007-2010)	7930 (girls 52.7%) children aged 9-14 years	0	Cross-sectional	The highest level of education among parents represented the parents' education level:	Overweight and obesity were defined by sex-age specific BMI according to the new growth charts for Taiwanese children and adolescents, which was based on WHO standards and health-related physical fitness records in Taiwan	n/a	Overweight:
			5	≤ 12			≤12 years: ref
				13–15			13–15 years: OR=0.9 (0.8-1.0)
				≥ 16 years			≥ 16 years: OR=0.7 (0.5-0.9)
				Questionnaire			
							Obesity:
							≤12 years: ref
							13–15 years: OR=0.9 (0.8-0.9)
							≥ 16 years: OR=0.5 (0.4-0.7)
Chen (2021) [82], China, conducted in Chongqing (2014)	17007 (girls 47.8%) children aged 6-12 years	0	Cross-sectional	Father's educational level:	Overweight and obesity were based on the sex-specific Centers for Disease Control and Prevention BMI-for-age growth charts	Adjust for age and gender	Overweight:
			7	~9 years			≤ 9 years: ref
				~12 years			~ 12: OR=1.234 (1.093-1.393)
				~15 years			~ 15: OR=1.255 (1.058-1.490
				>15 years			> 15: OR=1.154 (0.607-2.196)
				Questionnaire			

							Obesity:
							≤ 9 years: ref
							~ 12: OR=1.187 (1.020-1.381)
							~ 15: OR=1.296 (1.051-1.598)
							> 15: OR=1.094 (0.496-2.413)
Cook (2019) [83], US, National Health and Nutrition Examination Survey (2011-2016)	841 (girls 48.3%) children aged 6-19 years	0	Cross-sectional	Parental education level: Having a 4-year college or advanced degree	Overweight was defined by BMI of the Centers for Disease Prevention and Control	Adjusted for age, gender, nativity status, parental nativity status, and health insurance coverage	College/advanced degree: OR=0.65 (0.41-1.04)
			8	No college degree			No college degree: ref
				In home interview			
Ding (2021) [84], China, China Family Panel Studies (2010, 2012, 2014 and 2016)	6274 (girls 48.0%) children aged 0-15 years	2月6日	Cohort	Paternal or Maternal education:	Overweight and obesity were defined by BMI-for-age z-score with age-specific and sex-specific WHO growth reference	Adjusted for child sex, age, residence, survey year, average household income, paternal education level and maternal education level except itself	Father:
			7	Primary school or less			Primary school or less: ref
				Junior high school			Junior high school: OR=0.85 (0.75-0.97)
				Senior high school			Senior high school: OR=0.77 (0.64-0.92)
				Junior college or higher			Junior college or higher: OR=0.72 (0.55-0.93)
				Interviews			
							Mather:
							Primary school or less: ref
							Junior high school: OR=0.76 (0.67-0.86)
							Senior high school: OR=0.59 (0.47-0.72)
							Junior college or higher: OR=0.45 (0.34-0.60)
Donkor (2017) [85],	1895 (girls	5月6日	Cohort	Maternal education	Overweight and	Adjusted for birth	Overweight:

Norway, conducted in Oppland County	53.0%) children aged 5 years			above high school	obesity were defined by the sex-specific and age-specific BMI criteria (iso-BMI) of the International Obesity Task Force (IOTF)	weight SDS and maternal BMI	
			8	Paternal education above high school			Maximum high school: OR=1.75 (1.01-3.01)
				Both parent education above high school			Above high school: ref
				Parental questionnaire			
							Obesity:
							Maximum high school: OR=3.54 (1.20-10.42)
							Above high school: ref
Feng (2019) [86], China, China Health Nutrition Survey (2011)	1081 (girls 48.4%) children aged 7-18 years	0	Cross-sectional	Maternal education:	Overweight and obesity were defined using the classification criteria of body weight index for 5–19 years old boys and girls (z-scores): overweight if BMI > Mean + 1SD and obesity if BMI > Mean + 2SD	Adjust for children gender, maternal age, maternal employment status, and household income	Junior high school and below: ref
			7	Junior high school			High school: OR=2.17 (1.398-3.370)
				High school			Technical college: OR=2.397 (1.478-3.887)
				Technical college			College and above: OR=2.146 (1.293-3.560)
				College and above			
				Questionnaire			
Frye (2003) [87], German, Three consecutive surveys (1992–93, 1995–96, and 1998–99)	7611 children aged 5-14 years	0	Cross-sectional	Parental education (highest of father or mother)	Overweight and obesity were based on average centiles as suggested by Cole et al.	Adjusted for age, sex and survey	Overweight:
			7	Gathered from parents by a self-administered questionnaire			Less than 10 grades: ref
							10 grades: OR=1 (0.8-1.3)
							12 grades: OR=1 (0.7-1.3)

							University degree: OR=0.7 (0.5-0.9)
							Obesity:
							Less than 10 grades: ref
							10 grades: OR=1.1 (0.7-1.7)
							12 grades: OR= 0.7 (0.4-1.2)
							University degree: OR=0.4 (0.2-0.8)
Fuiano (2008) [88], Italy, conducted in San Marco in Lamis (initiated in 2005)	632 (girls 45.4%) children aged 3-8 years at baseline	2	Cohort	Parental educational level: Low with primary or lower secondary school degree	Overweight and obesity were defined by BMI according to Italian cross-sectional 2006 growth charts for Southern Italy	n/a	Paternal education level:
			4	High with upper secondary school or university degree			High: ref
							Low: OR=1.05 (0.71-1.54)
							Maternal education level:
							High: ref
							Low: OR=0.83 (0.56-1.21)
Grydeland (2012) [89], Norway, HEalth in Adolescents (HEIA) study (2007)	1103 (girls 50.3%) children aged 11 years	0	Cross-sectional	Parental education:	Overweight and obesity were defined by BMI with the International Obesity Task Force (IOTF) cut-off points	Adjusted for gender and puberty	≤12 years: ref
			8	≤12years			13-16 years: OR=0.58 (0.37-0.92)
				13-16 years			>16 years: OR=0.54 (0.33-0.89)
				>16 years			
				Internet-based questionnaire and a short paper questionnaire			
Gurzkowska	10950 (girls	0	Cross-	Maternal and	Overweight and	Adjusted for	Maternal education:

(2014) [90], Poland, "Elaboration of reference blood pressure ranges for children and adolescents in Poland" OLAF-PL0080 (OLAF) study	48.2%) children aged 7-18 years		sectional	Paternal education:	obesity were defined by BMI cutoffs according to the International Obesity Task Force (IOTF)	maternal/paternal education, income per capita, number of children	
			8	Primary			Primary: ref
				Vocational			Vocational:
				Secondary			boys OR=1.15 (0.84-1.58)
				University			girls OR=0.76 (0.57-1.02)
				Questionnaire			Secondary:
							boys OR=1.14 (0.82-1.57)
							girls OR=0.75 (0.55-1.01)
							University:
							boys OR=0.96 (0.67-1.39)
							girls OR=0.77 (0.54-1.11)
							Paternal education:
							Primary: ref
							Vocational:
							boys OR=1 (0.75-1.33)
							girls OR=0.86 (0.65-1.14)
							Secondary:
							boys OR=0.9 (0.66-1.23)
							girls OR=0.76 (0.55-1.04)
							University:
							boys OR=0.82 (0.56-1.20)
							girls OR=0.71 (0.47-1.05)
Gao (2022) [91], China, CHNS (1996-2000)	1883 children aged 7-18 years	4	Cohort	Parental education (highest attainment of parents) was classified into high (≥high school) and low (<high school) education groups.	Overweight was defined by WHO 2007 growth reference (BAZ>1SD)	Adjusted for age and sex	High: ref
			7				Low: OR=2.4 (1.7, 3.5)

Haas (2003) [92], US, Medical Expenditure Panel Survey Household Component (1996)	3775 children aged 6-11 and 12-17 years	0	Cross-sectional	Parental education:	Overweight was defined by the CDC's BMI-for-age charts	Adjusted for age, sex, ethnicity, country of birth, highest parental educational attainment, household income, receipt of AFDC and health insurance status single-parent household, and region	6-11 years
			8	12 years or less			12 years or less: OR=1.38 (1.05-1.82)
				More than 12 years			more than 12 years: ref
				Interview			
							12-17 years,
							12 years or less: OR= 1.32 (0.89-1.97)
							more than 12 years: ref
Herter-Aeberli (2019) [93], Switzerland (2002-2017/18)	2002 (n = 2493, girls 50.6%), 2007 (n = 2218, girls 51.2%), 2012 (n = 2963, girls 49.4%), and 2017/18 (n = 2279, girls 49.8%) children aged 6-12 years	0	Cross-sectional	The educational levels of both parents were combined and categorized into the following three groups:	Overweight and obesity were defined by the Centers for Disease Control and Prevention (CDC) reference values using the cut offs of the 85th and the 95th percentiles	Adjusted for parental origin, physical activity, and sex	Overweigh in 2017/18:
			8	Low (obligatory school time) Moderate (apprenticeship with or without professional maturity)			Low: OR=1.319 (0.662-2.630)
				High (university of applied sciences, technical university, or university)			Medium: OR=1.721 (1.245-2.377)
				Questionnaire			High: ref
							Obesity in 2017/18:
							Low: OR=3.118 (1.458-6.666)
							Medium: OR=1.945 (1.181-3.204)

							High: ref
Hoang (2018) [94], Vietnam, conducted in Hai Phong city (2016)	2334 (girls 50.5%) children aged 6-9 years	0	Cross-sectional	Mother education:	Overweight and obesity were defined by BMI-for age-z-score with the WHO classification	Adjusted for age, sex, mother's current employment status, and monthly household income	Above high school: ref
			8	Above high school			High school: OR=0.59 (0.44-0.81)
				High school			Below high school: OR=0.37 (0.26-0.53)
				Below high school			
				Self-administrated questionnaire			
Homs (2023) [95], Spain, The PASOS Study (2019-2020)	2791 children (girl: 51.9%) aged 8-16 years	0	Cross-sectional	Parental educational level was categorized into university and less than university	Obesity was defined as >WHO growth reference median + 2 standard deviation (SD)	Adjusted for gender, age, adherence to the Mediterranean diet, minutes of MVPA, and school	Parental education, obesity
			8				Low: ref
							High: OR=0.52 (0.39-0.69
Ikeda (2019) [96], Japan, Longitudinal Survey of Newborns in the 21st Century (the first survey took in 2001-2002)	15427 (girls 48.5%) children aged 42-66 months and 7-12 years	8.5	Cohort	Maternal education:	Overweight and obesity were defined by BMI with the International Obesity Task Force criteria (IOTF)	Adjusted for children's sociodemographic characteristics (sex, birth month, birthweight, gestational length, maternal educational background, regions and municipal types of residence, and living without siblings or with grandparents in the same household as markers of family structure), and behavioral characteristics	Age 42–66 months:
			7	Junior high school			Junior high school: OR=1.25 (0.87-1.80)
				High school			High school: OR=1.13 (0.97-1.31)
				Junior/career college			Junior/career college: ref

				University/higher Questionnaire		(weekday bedtime hours, time spent watching television per weekday, skipping breakfast as markers of sleep, sedentary behavior, and diet, and modes of travel to school)	University/higher: OR=1.00 (0.82-1.21)
							Age 7–12 years:
							Junior high school: OR=1.53 (1.16-2.02)
							High school: OR=1.25 (1.12-1.40)
							Junior/career college: ref
							University/higher: OR=1.06 (0.91-1.23)
Ip (2016) [97], China Hong Kong, Data from HKGS (2005–2006) and the Population By-census (2006)	14842 (girls 49.7%) children aged 6-19 years	0	Cross-sectional	Maternal education:	Overweight and obesity were defined by BMI with the International Obesity Task Force (IOTF) cut-off points	n/a	Prevalence of overweight and obesity: Secondary school or below: 17.15% and 3.84%
			5	Secondary school or below			Tertiary education or above: 15.03% and 3.00%
				Tertiary education or above			Children whose mother only completed secondary school or below had higher risk of childhood obesity (RR 1.41, 95%CI 1.13–1.76)
				Family questionnaire			

Inoue (2023) [98], US, National Health and Nutrition Examination Survey (1999–2018)	21,754 children (girl: 51.1) aged 6-17 years	0	Cross-sectional	Household education levels were defined using the education levels of the household reference person (i.e., the first household member aged ≥18 years listed on the household member roster, who owns or rents the residence where members of the household reside)	Obesity among children and adolescents was defined as age- and sex-specific BMI in the 95th percentile or greater based on the 2000 Centers for Disease Control and Prevention growth charts	Adjusted for age, gender, and race/ethnicity	College or above: ref
			8				Less than high school: RR=2.07 (1.81–2.37)
Juliusson (2010) [99], Norway, Bergen Growth Study (2003-2006)	6386 (girls 48.6%), 3793 (girls 49.5%) children aged 2-19 years assessed for association between parental education and overweight / obesity	0	Cross-sectional	Parental education:	Overweight and obesity were defined by BMI with the International Obesity Task Force (IOTF) cut-off points	Adjusted for age, sex, origin, increasing number of siblings, number of parents in the home, parental working situation	Secondary school not finished: OR=1.67 (1.1-2.52)
			8	Secondary school not finished			Secondary school finished: OR=1.48 (1.18-1.86)
				Secondary school finished			University education: ref
				University education			
				Parental questionnaire			
Keane (2012) [100], Ireland, First wave (2008) of the Growing Up in Ireland (GUI)	8136 (girls 48.7%) children aged 9 years	0	Cross-sectional	Maternal education:	Overweight and obesity were defined by BMI with the International Obesity Task Force (IOTF)	Adjusted for gender, Family type, Siblings, Equivalised household annual income, Parent weight status	Overweight:
			8	Third level education			Third level: ref
				Post secondary education			Post secondary: OR=1.18 (0.92–1.52)

study				Higher secondary education	cut-off points		Higher secondary: OR=1.11 (0.87–1.41)
				Lower secondary education or less			Lower secondary/less: OR=1.22 (0.91–1.64)
				Questionnaire			
							Obesity:
							Third level: ref
							Post secondary: OR=2.29 (1.47–3.55)
							Higher secondary: OR= 2.05 (1.35–3.11)
							Lower secondary/less: OR=2.7 (1.72–4.23)
Klein-Platat (2003) [101], France, conducted in Bas-Rhin (2001)	4326 (girls 48.6%) children aged 12.1±0.6 years	0	Cross-sectional	Father / Maternal education: Low (no formation, primary school or first years of secondary school)	Overweight was defined by BMI with the International Obesity Task Force (IOTF) cut-off points	Adjusted for mother's and father's obesity	Maternal education:
			8	Medium (secondary school or technical training)			Low: OR=1.621 (1.152-2.279)
				High (university)			Medium: OR=1.485 (1.071-2.057)
				Parental questionnaire			High: ref
							Paternal education:
							Low: OR=1.054 (0.764-1.453)
							Medium: OR=1.049 (0.756-1.455)
							High: ref
Ke (2023) [102], China, Jiangsu,	2127 children (girl: 58.01%)	0	Cross-sectional	Maternal education:	Overweight and obesity were defined	Adjusted for sex, age, grade,and ethnicity	Maternal educational level:

Anhui, Zhejiang, and Shanghai student survey (2020)	aged 9-17 years		8	Low education level (below elementary school, elementary school, and junior middle school)	by BMI-for-age z-scores with the WHO classification		Junior middle school: 1.47 (1.03, 2.11)
				Medium education level (high school or occupational school and college)			low: ref
				High education level (undergraduate or postgraduate and above)			
Lamerz (2005) [103], German, conducted in the City of Aachen (2001-2002)	1827 (girls 49.1%) children aged 5-7 years	0	Cross-sectional	Maternal/Paternal education:	Obesity was defined by BMI ≥ 90th percentile (German reference population based on Kromeyer-Hauschild et al.)	Adjusted for gender, maternal/paternal BMI, maternal/paternal education, maternal employment, paternal employment, m2 living space/person, single parent	Maternal education:
			8	13 years			13 years: ref
				10-12 years			10-12 years: OR=0.75 (0.45-1.26)
				9 years			9 years: OR=1.52 (0.88-2.62)
				No degree			No degree: OR=2.86 (1.36-6.03)
				Parental questionnaire			
							Paternal education:
							13 years: ref
							10-12 years: OR=1.37 (0.83-2.26)
							9 years: OR=1.3 (0.76-2.21)
							No degree: OR=1.69 (0.77-3.72)
Lasserre (2007) [104], Switzerland, conducted in Vaud (2005-2006)	5207 (girls 49.7%) children aged 12.3±0.5 years	0	Cross-sectional	Parents' education:	Overweight was defined by the sex- and age-specific BMI criteria of the International Obesity	Adjusted for age, sex, and each variable in the table	Primary: ref
			8	Primary			Secondary: OR=1.0 (0.7-1.4)
				Secondary			Tertiary: OR=0.7 (0.5-1.0)
				Tertiary			

				Questionnaire	Task Force (IOTF)		
Lazzeri (2014) [105], Italy, Italian Health Behaviour in School-aged Children 2010-HBSC	58928 (girls 50.3%) children aged 11-15 years	0	Cross-sectional	Parental education level:	Overweight was defined by BMI using the International Obesity Task Force (IOTF) cut-off values	Adjusted for parental education, breakfast consumption and students' residence area	Boys:
			8	Less than high school			Less than high school: OR=1.63 (1.38-1.91)
				High school			High school: OR=1.27 (1.10-1.48)
				University			University: ref
				Self-reported anonymous questionnaires			
							Girls:
							Less than high school: OR=2.07 (1.70-2.51)
							High school: OR=1/39 (1.14-1.70)
							University: ref
Le (2022) [106], Vietnam, conducted in Thanhhoa city (2021)	782 (girls 52.3%) children aged 6-11 years	0	Cross-sectional	Father/Mother education:	Overweight and obesity were defined by BMI-for-age z-scores with the WHO classification	n/a	Father education:
			6	Under secondary			Under secondary: OR=0.20 (0.03-0.69)
				Secondary			Secondary: OR=1.32 (0.77-2.26)
				High school			High school: ref
				University			University: OR=1.29 (0.92-1.83)
				Post university:			Post university: OR=1.23 (0.72-2.08)
				Self-administrated questionnaire			
							Mother education:
							Under secondary: OR=0.25 (0.04-0.92)
							Secondary: OR=0.96 (0.55-1.64)

							High school: ref
							University: OR=1.32 (0.93-1.87)
							Post university: OR=1.14 (0.63-2.03)
Lee (2020) [107], Korea, Korea National Health and Nutrition Examination survey (2007-2009, 2010-2012, 2013-2015)	14482 (girls 47.2%) children aged 2-18 years	0	Cross-sectional	Maternal/Paternal education level:	Overweight and obesity were defined using the age- and sex-specific BMI percentiles in reference to the 2007 Korean Growth Charts	Adjusted for maternal employment status, maternal smoking status, maternal BMI, paternal BMI, child's age, child's sex, monthly family income, and survey year	Survey years: 2007-2009
			8	Less than high school			Paternal education level:
				High school			Greater than high school: ref
				Greater than high school			Less than high school: OR=1.05 (0.55-2.01)
				Questionnaire			High school: OR=1.13 (0.72-1.76)
							Maternal education level
							Greater than high school: ref
							Less than high school: OR=1.47 (0.77-2.82)
							High school: OR=1.06 (0.71-1.60)
							Survey years: 2010-2012
							Paternal education level
							Greater than high school: ref
							Less than high school: OR=1.64 (0.76-3.50)
							High school: OR=1.09 (0.70-1.68) Maternal education level:
							Greater than high school: ref
							Less than high school: OR=0.76 (0.26-2.25)
							High school: OR=1.22 (0.79-1.87)
							Survey years: 2013-2015:
							Paternal education level

							Greater than high school: ref
							Less than high school: OR=1.13 (0.49-2.59)
							High school: OR= (0.86-2.05)
							Maternal education level
							Greater than high school: ref
							Less than high school: OR=1.10 (0.44-2.75)
							High school: OR=1.18 (0.75-1.86)
Li (2009) [108], China conducted in Xinjiang (2009)	310 children aged 6-13 years	0	Case-control	Father's education	Overweight and obesity	Adjusted for family history of obesity	Father education:
			8	Questionnaire	6 year: defined by weight-for-height with the WHO classification 7-17 years: BMI reference norm for screening overweight and obesity in Chinese children and adolescents		Illiterate: ref
							Primary school: OR=1.65 (0.29-9.26)
							Junior high school: OR=0.29 (0.23-1.04)
							Senior high school and vocational school: OR=0.36 (0.18-0.73)
							College and above: OR=0.80 (0.42-1.53)
Li (2021) [109], China, conducted in Guangdong	552 (girls 46.6%) children aged 3-7 years	0	Cross-sectional	Parental education:	Obesity was defined based on the classification of	Adjusted for only child and urban/rural	Parental education (low): OR=1.463 (1.202-2.122)
			8	Below university			Parental education (high): ref

				University and above	WHO (z-scores)		
				Questionnaire			
Liang (2018) [110], China, conducted in Shanghai (2018)	3789 children aged 3-6 years	0	Cross-sectional	Father's education	Overweight and obesity were defined by BMI percentiles with the WHO classification	n/a	Prevalence of overweight:
			6	Parental questionnaire			Primary school and below: 0
							Junior high school: 13.7%
							Senior high school: 14%
							College: 13.3%
							University: 15%
							Master's and above: 12.5%
							Prevalence of obesity:
							Primary school and below: 14.3%
							Junior high school: 18.9%
							Senior high school: 16.5%
							College: 18.8%
							University: 14.6%
							Master's and above: 9.7%
Liu (2016) [111], China, conducted in Guangzhou (2014)	9917 (girls 44.2%) children aged 5-12 years	0	Cross-sectional 7	Father's/Mother's education:	Overweight and obesity were defined by BMI-for-age z-score with the WHO reference 2007	Adjust for all social-demographic factors, and school as a random effect	Father education:
				Primary school and junior high school			Primary school and junior high school: ref
				Senior high school and vocational school			Senior high school and vocational: OR=1.11 (0.95-1.31)
				University and above			University and above: OR=0.89 (0.69-1.16)
				Parental questionnaire			
							Mother education:
							Primary school and junior high school: ref

							Senior high school and vocational: OR= 1.21 (1.03-1.43)
							University and above: OR=1.51 (1.16-1.97)
Liu (2018) [112], China, conducted in Shenyang (2017)	3670 (girls 49.0%) children aged 9-12 years	0	Cross-sectional	Father's/Mother's education:	Obesity was defined by the cut-off BMI recommended by Working Group on Obesity in China (WGOC)	Adjust for age, sex, residence area (urban/rural) and school (which school the children belonged to)	No or compulsory basic education: ref
			7	No or compulsory basic education			Father with higher education: OR=0.76 (0.55-1.04)
				Higher education			Mother with higher education: OR=1.26 (0.87-1.81)
				Parental questionnaire			
Lombardo (2015) [113], Italy, OKkio alla SALUT (2010)	42431 (girls 48.5%) children aged 8-9 years	0	Cross-sectional	Parents education (highest of one of parents):	Severe obesity was defined by the WHO and International Obesity Task Force (IOTF) criteria	Adjusted for gender, age, parents' nationality, parents' nutritional status, geographical area	WHO:
			8	University			University: ref
				High school			High school: OR=1.65 (1.33-2.04)
				Less than high school			Less than high school: OR=1.77 (1.41-2.22)
							IOTF:
							University: ref
							High school: OR=1.79 (1.36-2.37)
							Less than high school: OR=1.97 (1.45-2.69)
Mazur (2008) [114], Poland	4248 (girls 48.6%) children aged 10.4±2.4 years for girls and 10.5±2.3 years for boys	0	Cross-sectional	Father and mother education A questionnaire was distributed among all the parents of children	Obesity was defined by BMI using the International Obesity Task Force (IOTF) cut-off points	Adjusted for number of family members, financial status, father/mother education, mother's employment outside home	OR (95%CI) of obesity for girls, father education:
			8				elementary vs. vocational: OR=1.21 (0.86-1.70)
							elementary vs. secondary: OR=1.60 (1.1-2.32)

							elementary vs. university: OR=1.61 (0.89-2.93) (no significant difference in boys)
							OR (95%CI) of obesity for girls, mother education:
							elementary vs. vocational: OR=0.90 (0.52-1.30)
							elementary vs. secondary: OR=1 (0.7-1.44)
							elementary vs. university: OR=1.07 (0.6-1.89) (no significant difference in boys)
Milanovic (2020) [115], Croatia, WHO Europe Childhood Obesity Surveillance Initiative (2015/2016)	5591 (girls 49.7%) children aged 7-9 years	0	Cross-sectional	Maternal educational level: elementary school	Overweight and obesity were defined using the WHO criteria	Adjusted for age, sex, geographical region, level of urbanization and maternal employment	College degree or higher: ref
			8	high school			High school: OR=1.24 (1.08-1.42)
				college degree or higher			Elementary school: OR=0.94 (0.71-1.25)
				Parental questionnaire			
Moraeus (2012) [116], Sweden	3535 (girls 48.4%) children aged 7-9 years	0	Cross-sectional	Maternal education:	Overweight and obesity were defined by BMI with the International Obesity Task Force (IOTF) cut-off points	Adjusted for level of urbanization, Area education level, Origin parents, Parental weight status, Regular exercise (father), Artificially sweetened beverages, Breakfast on the day of study, Inactivity, TV/computer in	Overweight:
			8	>12 years			> 12 years: ref
				10-12 years			10–12 years: OR=1.59 (1.33-1.92)
				≤ 9 years			≤9 years: OR=1.87 (1.22-2.86)
				Questionnaire			
							Obesity:
							> 12 years: ref

						bedroom, Sports, Member of sports club	10–12 years: OR=1.48 (0.99-2.22)
							≤9 years: OR=2.25 (1.06-4.78)
Moschonic (2022) [117], Belgium, Bulgaria, Finland, Greece, Hungary, and Spain, Feel4Diabetes study (2016-2018)	12193 (girls 50.6%) children aged 8.20±1.01 years	0	Cross-sectional	Education of fathers/mothers:	Overweight and obesity were defined by BMI with the International Obesity Task Force (IOTF) cut-off points	Adjusted for sex, age of parents, education of parents, occupation of parents, employed part-time, parental weight status	Mother education:
			8	<9 years			>14 years: ref
				9-14 years			9-14 years: OR=1.56 (1.26-1.93)
				>14 years			< 9 years: OR=1.28 (0.88-1.86)
				Questionnaire			
							Father education:
							>14 years: ref
							9-14 years: OR=1.63 (1.31-2.03)
							< 9 years: OR=2.16 (1.54-3.05)
Moschonis (2010) [118], Greece, The Healthy Growth Study (initiated in 2007)	729 (girls 48.0%) children aged 9-13 years	0	Cross-sectional	Paternal/Maternal education:	Overweight and obesity were defined by BMI with the International Obesity Task Force (IOTF) cut-off points	n/a	Paternal education:
			6	≤9 years			≤9 years: ref
				9-14 years			9-14 years: OR=1.26 (0.78-1.92)
				>14 years			>14 years: OR=0.93 (0.59-1.46
				Face-to-face interview			
							Maternal education:
							≤9 years: ref
							9-14 years: OR=1.17 (0.74-1.80)
							>14 years: OR=1.13 (0.69-1.85)
Murer (2016) [119], Swiss	2724 (girls 49.5%) children aged 6-12 years	0	Cross-sectional	The educational levels of the parents were combined and categorized into the following three groups:	Overweight and obesity were defined by the age- and sex-specific BMI percentiles of the Centers for Disease	Adjusted for parental nationality, physical activity, media consumption, breakfast; age as a covariate	Boys

			8	Low (obligatory school time),	Control and Prevention (CDC)		Low:
				Moderate (apprenticeship with or without professional maturity),			overweight: OR=0.903 (0.428-1.905)
				High (university of applied sciences, technical university or university)			obesity: OR=1.019 (0.416-2.495)
				Questionnaire			Medium:
							overweight: OR=1.059 (0.710-1.579)
							obesity: OR=1.529 (0.870-2.686)
							High: ref
							Girls:
							Low:
							overweight: OR=1.566 (0.783-3.131)
							obesity: OR=1.510 (0.565-3.988)
							Medium:
							overweight: OR=1.505 (0.989-2.313)
							obesity: OR=2.517 (1.233-5.139)
							High: ref
Noh (2014) [120], Korea, Korean Survey on the Obesity of Youth and Children (2009)	9411 (girls 46.7%) children aged 10-18 years	0	Cross-sectional	Paternal/Maternal education:	Overweight was defined by BMI proposed by the Extended International Obesity Task Force (IOTF)	Adjusted for children's gender, education, region, parental interest in weight, family structure, parents working, economic	Paternal education:
			8	Middle school graduate or lower			Middle school graduate or lower: OR=1.3 (0.9-1.8)
				High school graduate			High school graduate: OR=1.1 (0.9-1.3)

				University graduate or higher<br>Questionnaire		status, parent's body shape	University graduate or higher: ref<br>Maternal education:<br>Middle school graduate or lower: ref<br>High school graduate: OR=0.9 (0.7-1.2)<br>University graduate or higher: OR=0.9 (0.7-1.3)
Noh (2014) [121], Korea, Korean Survey on the Obesity of Youth and Children (2009)	8555 (girls 45.0%) children aged 10-18 years	0	Cross-sectional<br>6	Father's/Mother's education:<br>Middle school graduate or less<br>High school graduated<br>University graduated or more	Overweight was defined by BMI proposed by the Extended International Obesity Task Force (IOTF)	n/a	Prevalence of overweight<br>Paternal education,<br>Middle school graduate or lower: 20.4%<br>High school graduate: 18.5%<br>University graduate or higher: 18.3%<br>Maternal education,<br>Middle school graduate or lower: 19.8%<br>High school graduate: 18.3%<br>University graduate or higher: 19.1%
Padez (2005) [122], Portugal (2002-2003)	4511 (girls 50.4%) children aged 7-9.5 years	0	Cross-sectional<br>8	Paternal/Maternal education:<br>Primary<br>Secondary<br>University<br>Parental	Overweight and obesity were defined by the age- and sex-specific BMI cut-off points of the International Obesity	Adjusted for sex, maternal obesity and level of education, and birthweight	Paternal education:<br>Primary: ref<br>Secondary:<br>Overweight: OR=1.15 (1.08-1.21)<br>Obesity: OR=0.91 (0.86-0.95)

				questionnaire	Task Force (IOTF)		
							University:
							Overweight: OR=0.92 (0.86-0.97)
							Obesity: OR=0.42 (0.39-0.44)
							Maternal education:
							Primary: ref
							Secondary:
							Overweight: OR=1.1 (1.04-1.15)
							Obesity: OR=1.13 (1.08-1.17)
							University:
							Overweight: OR=0.96 (0.9-1.01)
							Obesity: OR=0.56 (0.53-0.58)
Paduano (2020) [123], Italy, conducted in Modena (2018)	588 (girls 46.8%) children aged 6-7 years	0	Cross-sectional	Mother, Father and parents education:	Overweight and obesity were defined by BMI with the International Obesity Task Force (IOTF)	n/a	Prevalence of overweight and obesity
			6	< High school			Mother education:
				High school			< High school: 31.6%
				University			High school: 25.9%
				A self-administered questionnaire was distributed to the parents			University: 20.2%
							Father/ education:
							< High school: 29.7%
							High school: 27.8%
							University: 13.1%
							Parents education:
							< High school: 34.2%
							High school: 28.2%

							University: 22.1%
Patsopoulou (2015) [124], Greece, Feeding Exercise Trial in Adolescents (FETA)	816 (girls 55.9%) children aged 12-18 years	0	Cross-sectional	Maternal education	Overweight and obesity were defined by BMI with the criteria suggested by Cole et al.	Adjusted for age, gender, BMI for mothers, when your child ask to eat/does he or she claim to be hungry, how often do you eat in the following rooms (bedroom, living room), PIMCQ-2 score of father (worry)	Secondary school: OR=3.39 (1.61-7.12)
			8	Parental questionnaire			High school: OR=1.58 (0.92-2.70)
							University: ref
Pham (2019) [125], Vietnam, conducted in Hanoi (2014)	821 (girls 54.5%) children aged 11-12 years	0	Cross-sectional	Father and mother education leve	Overweight and obesity were defined by BMI with the WHO standard cut-offs	Adjusted for family characteristics, child characteristics, physical activity and lifestyle behaviors of children	Father education:
			7	Primary			Primary: ref
				Secondary			Secondary: OR=0.72 (0.12- 4.32)
				College or university			College or university: OR=0.65 (0.42-1.00)
				Parental questionnaire			
							Mother education:
							Primary: ref
							Secondary: OR=1.14 (0.30-4.31)
							College or university: OR=0.63 (0.41-0.97)

Saha (2022) [126], India, National Family Health Survey-4 (2015-2016)	176225 (girls 48.7%) children aged 0-59 months	0	Cross-sectional	Mother's level of education: Illiterate	Overweight and obesity were defined by BMI Z-scores with the WHO classification	Adjusted for sex of the child, the child's age, currently breastfeeding, birth rank, mother's educational level, age at marriage, mother's BMI, place of residence, region, social group, religious beliefs, wealth quintile, and dietary diversity score	Illiterate: ref
			8	Primary			Primary: RR=0.98 (0.88-1.09)
				Secondary			Secondary: RR=0.93 (0.85-1.02)
				Higher			Higher: RR=1.11 (0.98-1.26)
Santiago (2012) [127], Spain, "Alimenta su salud" (2008)	3101 (girls 49.4%) children aged 6-12 years	0	Cross-sectional	Father's education:	Overweight and obesity were defined by BMI with the International Obesity Task Force (IOTF) cut-off points	Adjusted for age, birth weight, number of siblings, father obese, mother obese	Father education level
			8	None or primary			boys:
				Secondary school or university degree			none or primary school: ref
				Questionnaire			secondary school or university degree: OR=0.8 (0.6-0.9)
							girls:
							none or primary school: ref
							secondary school or university degree: not significant (data not shown)
Schooling (2010) [128], China Hong Kong, "Children of 1997" birth cohort (initiated in	7108 (girls 47.2%) children aged 6-11 years	11	Cohort	Parental education:	Overweight and obesity were defined by BMI with the International Obesity Task Force (IOTF)	Adjusted for sex, mother's birthplace, neighborhood median income	7 years:
			7	≤9th grade			≤9th grade: ref
				10th-11th grade			10th to 11th grade: OR=0.97 (0.85-1.18)
				≥12th grade			≥12th grade: OR=0.89 (0.73-1.09)

1997)				Questionnaire and hardcopy Maternal and Child Health Centres records	cut-off points		
							11 years:
							≤9th grade: ref
							10th to 11th grade: OR=0.98 (0.86-1.13)
							≥12th grade: OR=0.78 (0.66-0.92)
Schule (2016) [129], German, Data were pooled from three surveys (2004 and 2007)	3499 (girls 47.0%) children aged 5-7 years	0	Cross-sectional	The highest level of completed education achieved either by the mother or the father was considered.	Overweight was defined by BMI using the International Obesity Task Force (IOTF) cut-off values	Adjusted for individual parental and child factors, perceived exposures and housing characteristics, contextual neighborhood characteristics	Low: OR=1.93 (1.38-2.69)
			8	‘High’ included a final degree at university or technical college, A-levels, or advanced technical college entrance qualification			Medium: OR=1.53 (1.14-2.05)
				‘Middle’ included upper secondary school certificate or adequate graduation			High: ref
				‘Low’ included a lower secondary school certificate or no graduation			
				Parental questionnaire			
She (2015) [130],	2431 (girls	0	Cross-	Parental education	Overweight and	Adjusted for parental	Father:

China conducted in Sichuan	44.1%) children aged 7-12 years		sectional		obesity were defined by the BMI reference norm for screening overweight and obesity in Chinese children and adolescents	occupation, parental education, monthly family income	
			7	Parental questionnaire			Primary school and below:
							Overweight: OR=0.95 (0.46-2.15)
							Obesity: OR=0.86 (0.23-1.58)
							Junior high school:
							Overweight: OR=0.56 (0.44-1.56)
							Obesity: OR=0.75 (0.67-2.04)
							Senior high school:
							Overweight: OR=0.21 (0.46-2.34)
							Obesity: OR=0.49 (0.4-2.51)
							University and above: ref
							Mother:
							Primary school and below:
							Overweight: OR=1.43 (0.21-2.05)
							Obesity: OR=1.37 (0.22-1.98)
							Junior high school:
							Overweight: OR=0.79 (0.33-1.66)
							Obesity: OR=0.82 (0.27-2.34)
							Senior high school:
							Overweight: OR=0.56 (0.28-2.64)
							Obesity: OR=0.54 (0.1-0.91)
							University and above: ref

Singh (2008) [131], US, National Survey of Children's Health (2003)	46707 (girls 48.5%) children aged 10-17 years	0	Cross-sectional	Highest household or parental education level	Obesity was defined by gender- and age-specific 95th percentile BMI cut-off points from the 2000 Centers for Disease Control and Prevention growth charts	Adjusted for age, gender, household composition, place of residence, language use, household education or poverty status, social capital, perceived neighborhood safety, and behavioral factors	<12: OR=1.42 (1.12–1.81)
			8	Computer-assisted telephone interviewing			12: OR=1.42 (1.27–1.58)
							13+: ref
Sun (2015) [132], China Nanjing, Jintan Child Health Project in China (2004-2007)	136 (girls 44.0%) children aged 2-4 years	3	Cohort	Father /Mother education: Lower than middle school High school	Overweight and obesity were defined by BMI with the WHO age- and sex-specific standards (2006)	n/a	Prevalence of overweight and obesity
			7	College or higher			Father education:
				Self-administered questionnaire			Lower than middle school: 20%
							High school: 19%
							College or higher: 25.4%
							Mother education:
							Lower than middle school: 26.2%
							High school: 14.9%
							College or higher: 25.5%
Veldhuis (2013) [133], Netherlands, "Be active, eat right" study (2007-2008)	5582 (girls 49.4%) children aged 5 years	0	Cross-sectional	Maternal education:	Overweight and obesity were defined by BMI with the International Obesity Task Force (IOTF)	Adjusted for maternal age, relevant mediating characteristics of the mother (weight status, watching TV), relevant	Overweight:
			8	High level (academic higher education/university			High: ref

				education, higher professional education),	cut-off points	mediating characteristics of the child (consuming breakfast, watching TV)	
				Mid level (pre-university education, senior general secondary education, and senior secondary vocational education),			Medium: OR=1.36 (1.05-1.77)
				Low level (preparatory secondary vocational education, lower secondary vocational education, primary education, and no education)			Low: OR=1.81 (1.33-2.46)
				Questionnaire			
							Obesity:
							High: ref
							Medium: OR=1.19 (0.64-2.18)
							Low: OR=2.84 (1.52-5.29)
Veltsista (2020) [134], Greece, Data were derived from 2 follow-ups of the Greek 1983 National Perinatal Survey (1990, 2001)	7219 7-year-olds (girls 48.1%) and 2,842 18-year-olds (girls 54.6%)	11	Cohort	Mother / Father education: Up to 6 years,	Overweight and obesity were defined by BMI with the International Obesity Task Force (IOTF) cut-off points	Adjusted for gender	Mother education:
			8	7-12 years,			up to 6 years: ref
				More than 12 years			7-12 years:
				Parental questionnaire			7 years old: OR=1.03 (0.89-1.19)
							18 years old: OR=1.25 (0.88-1.79)
							>12 years:

							7 years old: OR=0.97 (0.80-1.16
							18 years old: OR=0.93 (0.59-147)
							Father education:
							up to 6 years: ref
							7-12 years:
							7 years old: OR=1.1 (0.95-1.28)
							18 years old: OR=1.11 (0.77-1.61)
							>12 years:
							7 years old: OR=1.22 (1.03-1.46),
							18 years old: OR=1.15 (0.75-1.77)
Wang (2017) [135], China, conducted in Hong Kong (2015)	894 (girls 49.6%) children aged 9-12 years	0	Cross-sectional	Maternal educational level:	Overweight and obesity were defined by BMI with the International age- and sex-specific cutoff points	Adjusted for age, gender, family related variable, children's early life development-related variables, and children's behavioral variables	Below college: ref
			8	Below college			College or above: OR=0.52 (0.29, 0.93)
				College or above			Undisclosed: OR=2.30 (0.47, 11.27)
				Undisclosed			
				Parental questionnaire			
Wu (2018) [136], China, conducted in Shanghai (2016-2017)	1122 (girls 46.7%) children 7-15 years	0	Cross-sectional	Maternal education:	Obesity was defined by the BMI norm for screening overweight and obesity in Chinese children and adolescents	Adjusted for gender, monthly family income, maternal age, parental obesity, birth weight, sleeping time, intake of greasy food and sweets, appetite	Junior high school and below: OR=1.255 (1.022, 1.514)
			8	Junior high school and below			Technical secondary school or Senior high school and above: ref
				Technical secondary school or Senior high school and above			
				Parental questionnaire			

Xu (2016) [137], Central China, conducted in Nanchang from Jiangxi Province, Wuhan from Hubei Province, and Changsha from Hunan Province	4644 (girls 45.7%) children aged 7-12 years	0	Cross-sectional<br>8	Father and mother education:<br>Primary school or lower<br>Junior high school<br>Senior high school<br>College or above<br>Questionnaire	Overweight and obesity were defined as per Application of Overweight Obesity in BMI Screening Criteria into Chinese Students	Adjust for parental occupation, parental education level, family monthly income, lifestyle behaviors (daily exercise, snacking per month, sodas drinking per week)	Father education (overweight and obesity):<br>Primary school or lower: ref<br>Junior high school:<br>Overweight: OR=0.69 (0.13, 3.72)<br>Obesity: OR=0.75 (0.44, 1.27)<br>Senior high school:<br>Overweight: OR=0.67 (0.49, 0.92)<br>Obesity: OR=0.66 (0.44, 1)<br>College or above:<br>Overweight: OR=0.24 (0.09, 0.67)<br>Obesity: OR=0.54 (0.32, 0.9)<br><br>Mother education:<br>Primary school or lower: ref<br>Junior high school:<br>Overweight: OR=0.41 (0.15, 1.14)<br>Obesity: OR=0.63 (0.34, 1.18)<br>Senior high school:<br>Overweight: OR=0.79 (0.31, 1.98)<br>Obesity: OR=0.36 (0.15, 0.89)<br>College or above:<br>OR=0.28 (0.18, 0.43)<br>Obesity: OR=0.21 (0.08, 0.55)
Yi (2012) [138], China, conducted in Xi'an (2008-2009)	516 children aged 7-18 years	0	Case-control<br>7	Maternal education:<br>"high" means the mother received advanced education	Obesity was defined by BMI using the Working Group on Obesity in China (WGOC) references	Adjusted for sleeping time, watching TV/playing video games/using computers, parental overweight	High maternal education: OR=0.148 (0.074, 0.296)<br>Low maternal education: ref

				Parental questionnaire			
Zhai (2018) [139], China, conducted in Chengdu (2013)	853 (girls 52.4%) children aged 7-12 years	0	Cross-sectional	Maternal/Paternal education:	Overweight and obesity were defined by BMI with the Working Group on Obesity in China	Adjust for age, maternal BMI, birth weight, schools, family average annual income, paternal/maternal education, maternal occupation and paternal occupation	Maternal education:
			8	≤Primary school			Boys:
				Middle/secondary school			≤Primary school: ref
				≥College education			Middle/secondary school: OR=0.82 (0.30, 2.51)
				Questionnaire			≥College education: OR=1.17 (0.34, 4.34)
							Girls:
							≤Primary school: ref
							Middle/secondary school: OR=1.46 (0.44, 6.75)
							≥College education: OR=0.65 (0.18, 2.80)
Zhang (2015) [140], China, conducted in Jiangxi (2013)	2862 (girls 41.8%) children aged 8.42±1.02 years	0	Cross-sectional	Maternal education:	Overweight and obesity were defined by BMI with the Working Group on Obesity in China	Adjusted for birth weight, whether the only child, weekly meat >3 days, food preference for vegetables and fruits	College and above: OR=1.485 (1.016, 2.170)
			8	Below college			
				College and above Questionnaire			Paternal education:
							Boys:
							≤Primary school: ref
							Middle/secondary school: OR=0.48 (0.11, 2.04)
							≥College education: OR=0.63 (0.13, 3.00)
							Girls:
							≤Primary school: ref
							Middle/secondary school: OR=1.62 (0.38, 8.75)

							≥College education: OR=0.89 (0.21, 4.31)
							College and above: OR=1.485 (1.016, 2.170)
Deng (2016) [141], China, conducted in Hunan	2012: 109,129 (girls 49.2%)	0	Cross-sectional	Before and after the Nutrition Improvement Program for Compulsory Education Students in Rural Area	Overweight and obesity were defined by BMI with the criteria of the Working Group on Obesity in China (WGOC)	n/a	Rate of overweight [n (%)]
	2015: 115,581		5				After/Before
	(girls 43.9%) children aged 6-14 years						2012: 7.8 (8536/109,129)
							2015: 9.5 (11035/115,581)
							Rate of obesity [n (%)]
							After/Before
							2012: 4.0 (4314/109,129)
							2015: 5.6 (6521/115,581)
Ding (2023) [142], China, Beijing, Shanghai, Nanjing, Chengdu, and Xi'an cohort study (2015)	1691 children (girl: 49.21%) aged 11.5±2.1 years old	2	Cohort	School cafeteria food policy	Overweight and obesity were defined based on the Chinese national standard, the "WS/T 586–2018 Screening for overweight and obesity among school-aged children and adolescents" published by the National Health Commission of the People's Republic of China	Adjusted for age (years), sex, school level (primary and middle school), location, pocket money, and survey year.	Yes: OR=0.33 (0.17, 0.63)
			8				No: ref.
Fu (2018) [143],	113,322 (48.2%	7	Cohort	School awarded or	Obesity (including	n/a	Rate of obesity [n (%)]

China Hong Kong, Student Health Service (SHS) (2007/2008-2013/2014)	girls) children aged 9.5 years		8	not awarded with the EatSmart School Accreditation Scheme (ESAS)	overweight) was defined as body weight exceeding 120% of the median weight-for-height derived from HK reference data		Awarded: 19.4 (864/4451)<br>Not awarded: 20.7 (42991/207,789)
Kenney (2020) [144], US, National Survey of Children's Health (2003-2018)	173,013 children aged 10-17 years	0	Cross-sectional<br>8	Before and after the Healthy, Hunger-Free Kids Act of 2010 (HHFKA)	Obesity was defined as having a body mass index (BMI) above the ninety-fifth percentile for a child's age and biological sex; Overweight: from the eighty-fifth to below the ninety-fifth percentiles according to the 2000 Growth Charts of the Centers for Disease Control and Prevention.	Adjusted for age, biological sex, race/ethnicity (non-Hispanic white, non-Hispanic black, Hispanic/Latino, and non-Hispanic other), and the poverty status of the household (at or below 100 percent of the federal poverty level)	Rate of overweight [n (%)]<br>Pre-HHFKA：<br>2003: 15.8 (6460/42,147)<br>Post-HHFKA:<br>2018: 15.7 (2258/15,427)<br>Rate of obesity (%)<br>Pre-HHFKA：<br>2003: 14.8 (5843/42,147)<br>Post-HHFKA:<br>2018: 15.1 (2029/15,427)
Leung (2012) [145], US, National Health and Nutrition Examination Survey (NHANES)	5193 (girls 51.3%) children aged 4-19 years	0	Cross-sectional	Participant or not participant in the Supplemental Nutrition Assistance Program (SNAP)	Overweight and obesity:	Adjusted for age, gender, race/ethnicity, HR place of birth, HR education level, HR marital status, household size, health insurance, poverty	Rate of overweight [n (%)]

(1999–2008)			8		For children aged 4 to 17 years, BMI was transformed into z scores by using the 2000 Centers for Disease Control and Prevention Growth Charts and categorized into normal weight, overweight, or obese by using established age- and gender-specific cut points. For children aged 18 to 19 years, BMI was classified into standard weight categories	income ratio, household food security, and participation in other food assistance programs	SNAP Nonparticipants: 21.6
							(813/3780)
							SNAP Participants: 18.7 (270/1342)
							Rate of obesity [n (%)]
							SNAP Nonparticipants: 14.9
							(649/3780)
							SNAP Participants: 17.5 (254/1342)
Li (2016) [146], China, conducted in Yunnan	2012-2013：15,199	0	Cross-sectional	Before and after the Nutrition Improvement Program for Compulsory	Overweight and obesity were defined by BMI according to the growth standards of China "Screening	n/a	Rate of overweight and obesity [n (%)]
	2013-2014：26,491		5				2012-2013: 10.07 (1527/15199)
	2014-2015：						2014-2015: 10.47 (3798/36274)

	36,274 children aged 6-18 years			Education Students in Rural Area	for overweight and obesity among school, age children and adolescents”		
Øvrebø (2022) [147], Norway, the Norwegian Childhood Growth Study (NCGS) and Norwegian Youth Growth Study (NYGS) (2010)	8427 children aged 8.5 years old	2.5	Cohort	Participant or not participant in the fruit and Vegetable policy	Outcomes were BMI and overweight including obesity (OW/OB) in the third (age approximately 8.5 years) and eighth grade (age approximately 13 years), and WC and waist to height ratio (WtHR) in the third grade	Adjusted for parental education level	Boy: 1.85 (0.82, 4.17)
			8				Girl: 0.78 (0.33, 1.86)
							No: ref
Rito (2013) [148], Portugal, conducted in five Portuguese regions (2009)	266 (girls 52.6%) children aged 6-10 years	0	Pre-post	Before and after the Program Obesity Zero (POZ)	Overweight was defined as the BMI-for-age was ≥85th percentile, pre-obese and obese if the BMI-for-age was ≥85th and < 95th and ≥95th percentile, respectively. The	n/a	Rate of obesity [n (%)]
			9		75th percentile was used as the cut-off point for risk of		Baseline: 59.4 (158/266)
					abdominal obesity using Fernandez et		6 months: 50.3 (100/199)

					al.'s reference		
Sekhobo (2010) [149], US, Special Supplemental Nutrition Program for Women, Infants, and Children (WIC) (2002–2007)	2002:180,123<br>2003:187,766<br>2004:198,769<br>2005:202,945<br>2006:200,710<br>2007:198,633<br>(girls 49.5 %-49.9 %) children aged 2-5 years	0	Cross-sectional<br>6	Before and after the Special Supplemental Nutrition Program for Women, Infants, and Children (WIC)	Overweight was defined as sex-specific body mass index (BMI)-forage ≥85th and <95th percentile and obesity as sex-specific BMI-for-age ≥95th percentile	n/a	Rate of overweight [n (%)]<br>2002: 15.9 (28640/180123)<br>2007: 17.4 (35462/198633)<br>Rate of obesity (%)<br>2002: 16.5 (29720/180123)<br>2007: 14.7 (29199/198633)
Sekhobo (2014) [150], US, Nutrition Program for Women, Infants, and Children (WIC) (2004-2010)	110,773 children aged 3-4 years	0	Ecological study<br>7	Before and after the New York City 47 regulations	Obesity was defined as BMI for age at or below the 95th percentile on the basis of the 2000 CDC sex-specific growth charts	n/a	Rate of obesity (%)<br>High-Risk Neighborhoods:<br>Bronx:<br>2004–2006: 19.1%<br>2008–2010: 17.1%<br>Brooklyn:<br>2004–2006: 15.7%<br>2008–2010: 14.8%<br>Manhattan:<br>2004–2006: 18.6%<br>2008–2010: 15.3%<br>Low-Risk Neighborhoods:<br>Bronx:<br>2004–2006: 17.4 %<br>2008–2010: 16.1%<br>Brooklyn:<br>2004–2006: 13.6%<br>2008–2010: 12.8%

							Manhattan:
							2004–2006: 12.0 %
							2008–2010: 11.5%
Seo (2012) [151], US, conducted in Indiana (2008-2009)	246 (girls 65.5%) children aged 13.9±1.9 years	0	Cross-sectional	The presence of school nutrition policy of not	Overweight and obesity were defined when child-report-based BMI was at or higher than the 85th percentile for their age and sex	Adjusted for child's sex, grade, and race, and parent's education, household income, and employment status	Rate of overweight and obesity (%)
			8				Child's school allows students to purchase junk foods:
							Yes: 27.7%
							No: 27.1%
							Don't know: 41.7%
							Child's school allows students to purchase soda pop, sports drinks, or fruit drinks that are not 100% juice:
							Yes: 31.1%
							No: 19.2%
							Don't know: 37%
							Child's school allows students to purchase salty snacks that are not low in fat, such as regular potato chips or cheese puffs:
							Yes: 33.0%
							No: 23.6%
							Don't know: 28.8%
Sirikulchayanonta (2022) [152], Thailand, conducted in Bangkok (2004-2019)	5126 children aged 6-12 years	0	Pre-post	Before and after the Bright and Healthy Thai Kid project	Obesity was defined by	n/a	Rate of obesity [n (%)]
			10		weight for height (WFH) according to the standards itemized in the Institute of Nutrition Research, Mahidol University (INMU)		Before: 21.0 (889/4233)

					Tai Growth Program		After: 20.5 (1072/5230)
Kreider (2012)[153], US, National Health and Nutrition Examination Survey (NHANES, 2001-2006)	4418 Children aged 2-17 years	0	Cross-sectional 7	Participant or not participant in the Supplemental Nutrition Assistance Program (SNAP)	Obesity was defined by BMI z scores by using the 2000 Centers for Disease Control and Prevention Growth Charts and categorized into normal weight, obesity by using established age- and gender-specific cut points	n/a	Rate of obesity<br>SNAP Participants: 0.191±0.014<br>SNAP Nonparticipants: 0.179±0.012
Simmons (2012)[154], US, National Health and Nutrition Examination Survey (NHANES, 2008-2010)	385 children aged 4.2 years ±6 month	0	Cross-sectional 8	Participant or not participant in the Supplemental Nutrition Assistance Program (SNAP)	Obesity was defined as BMI for age at or below the 95th percentile on the basis of the 2000 CDC sex-specific growth charts	n/a	Rate of obesity<br>SNAP Participants: 20.5%<br>SNAP Nonparticipants: 15.3%

REFERENCE

[1] BEL-SERRAT S, HEINEN M M, MEHEGAN J, et al. Predictors of Wweight Status in School-aged Children: A Prospective Cohort Study [J]. European journal of clinical nutrition, 2019, 73(9): 1299-306.

[2] CHANG H, YANG L, LI L, et al. Obesity and Associated Factors among Primary and Middle School Students in Shenyang Urban Area [J]. Chinese Journal of School Health, 2012, 33(01): 118-9.

[3] CHEN W. Regression Analysis of Dietary Factors of Overweight in Preschool Children [J]. Maternal and Child Health Care of China, 2007, (12): 1676-7.

[4] CHEN Q, ZHANG R, GUO S. Influences of Family Feeding Mode on Obesity among Children and Adolescents [J]. Chinese Journal of Health Education, 2017, 33(11): 1024-7.
[5] CHEN T, FAN Y, SONG X, et al. Analysis of Prevalence and Risk Factors of Overweight and Obesity among Children and Adolescents in Jiangxi Province [J]. Chinese Journal of School Health, 2021, 42(11): 1724-7.
[6] DUDAS R A, CROCETTI M. Association of bicycling and childhood overweight status [J]. Ambulatory pediatrics : the official journal of the Ambulatory Pediatric Association, 2008, 8(6): 392-5.
[7] DUPUY M, GODEAU E, VIGNES C, et al. Socio-demographic and lifestyle factors associated with overweight in a representative sample of 11-15 year olds in France: results from the WHO-Collaborative Health Behaviour in School-aged Children (HBSC) cross-sectional study [J]. BMC public health, 2011, 11: 442.
[8] FLORES G, LIN H. Factors predicting severe childhood obesity in kindergarteners [J]. International journal of obesity (2005), 2013, 37(1): 31-9.
[9] GUAN Q, LIANG Z, MO Y. The prevalence and influencing factors of overweight and obesity in adolescents in Foshan city [J]. Journal of Tropical Medicine, 2018, 18(10): 1372-7.
[10] GUO Y, XU Q, KONG Y, et al. Effects of dietary and exercise habits on overweight and obesity of preschool children [J]. Maternal and Child Health Care of China, 2011, 26(36): 5709-12.
[11] TANG K H, NGUYEN H H, DIBLEY M J, et al. Factors associated with adolescent overweight/obesity in Ho Chi Minh city [J]. International journal of pediatric obesity : IJPO : an official journal of the International Association for the Study of Obesity, 2010, 5(5): 396-403.
[12] HUANG P, LI H, XU S. Analysis on the effect of formation of dietary and behavioral habits on childhood obesity in a region of Sichuan province [J]. Maternal and Child Health Care of China, 2017, 32(02): 355-7.
[13] JIA L. Prevalence of overweight and obesity in primary school students from Wenzhou city and the analyses of the risk factors [D]; Zhejiang University, 2013.
[14] KOLMAGA A, TRAFALSKA E, SZATKO F. Risk factors of excessive body mass in children and adolescents in Łódź [J]. Roczniki Panstwowego Zakladu Higieny, 2019, 70(4): 369-75.
[15] LI G. The Prevalence of Overweight and Obesity in Preschoolers in Fujian Province and Its Association with Circ RNAs [D]; Fujian Medical University, 2021.
[16] LU D, GUO H, LIU Y, et al. Analysis on the obesity and lifestyle of school-age children in Changping district, Beijing in 2017 [J]. Chinese Journal of Child Health Care, 2018, 26(12): 1354-7.
[17] LUO S, YAN S, HOU Y, et al. Relationship between eating habits and overweight and obeisity in school-age children [J]. Journal of Xinxiang Medical University, 2020, 37(02): 148-51.
[18] NIE G. Epidemiological investigation of simple obesity in urban and rural preschool children [J]. Maternal and Child Health Care of China, 2014, 29(13): 2062-4.
[19] PENGPID S, PELTZER K. Overweight, Obesity and Associated Factoes among 13-15 Years Old Students in the Association of Southeast Asian Nations Mamber Countries, 2007-2014 [J]. The Southeast Asian journal of tropical medicine and public health, 2016, 47(2): 250-62.
[20] QIN Y. Analysis on childhood obesity incidences between urban and rural in Chongqing and its related factors [D]; Chongqing Medical University, 2013.
[21] QIU L. Relationship between family feeding methods and obesity among children and adolescents in economically underdeveloped areas， Henan [J]. Modern Preventive Medicine, 2020, 47(05): 827-9+927.
[22] SALAHUDDIN M, PéREZ A, RANJIT N, et al. Predictors of Severe Obesity in Low-Income, Predominantly Hispanic/Latino Children: The Texas Childhood Obesity Research Demonstration Study [J]. Preventing chronic disease, 2017, 14: E141.
[23] SANTIAGO S, ZAZPE I, MARTí A, et al. Gender differences in lifestyle determinants of overweight prevalence in a sample of Southern European children [J]. Obesity research

& clinical practice, 2013, 7(5): e391-400.
[24] WANG Y. Analysis the influence factors of overweight and obesity about city school-age children [D]; Wuhan University of Technology, 2016.
[25] WEI H, LI B. Analysis on situation and relative risk factors of overweight and obesity among children in Longgang district of Shenzhen city [J]. Modern Preventive Medicine, 2009, 36(15): 2853-4+65.
[26] WU F, GAO J, TANG F, et al. Survey on the relationship between diet, physical activity and weight among middle school students in Jinshan District, Shanghai [J]. Chinese Journal of Health Education, 2019, 35(04): 323-7+45.
[27] XIANG M, YAO S. Epidemiological survey of simple obesity in preschool group children in urban areas of Xining City [J]. Maternal and Child Health Care of China, 2011, 26(23): 3608-9.
[28] YANG T, ZHANG K, GAO S, et al. Obesity and associated factors among students in Inner Mongolia in 2019 [J]. chinese Journal of School Health, 2021, 42(04): 611-4.
[29] ZENG Y, CHEN Z. Prevalence of overweight and obeisity and its influencing factors among primary school students in Yangchun [J]. Chinese Journal of School Health, 2010, 31(07): 835-6.
[30] ZHANG J, WEI M, YAO G, et al. The ststus and variation tendency of prevalence of simple obesity among the children aged 0-6 years old in Shangha [J]. Maternal and Child Health Care of China, 2011, 26(13): 1996-9.
[31] ZHANG Q, TAN X, YU L, et al. Status and influencing factors of overweight and obesity among school-sge children in Shandong urban areas [J]. Chinese Journal of Child Health Care, 2013, 21(05): 528-31.
[32] ZHANG C, DING X, FENG L, et al. Influences of family feeding and dietary habits on childhood obesity [J]. Chinese Journal of School Health, 2013, 34(09): 1056-8.
[33] ZOU X. Current status of the Middle school students obesity and its Association with Partial Fat Behaviour in Wuhanv [D]; Wuhan Sports University, 2020.
[34] ZURRIAGA O, PéREZ-PANADéS J, QUILES IZQUIERDO J, et al. Factors associated with childhood obesity in Spain. The OBICE study: a case-control study based on sentinel networks [J]. Public health nutrition, 2011, 14(6): 1105-13.
[35] BECK A L, TSCHANN J, BUTTE N F, et al. Association of beverage consumption with obesity in Mexican American children [J]. Public health nutrition, 2014, 17(2): 338-44.
[36] MARTIN-CALVO N, MARTíNEZ-GONZáLEZ M A, BES-RASTROLLO M, et al. Sugar-sweetened carbonated beverage consumption and childhood/adolescent obesity: a case-control study [J]. Public health nutrition, 2014, 17(10): 2185-93.
[37] CAO H, XU L. Detection rate and influencing factors of simple obesity in collective children aged 3-7 years in Urumqi [J]. Chinese Journal of Child Health Care, 2008, (05): 590-1.
[38] DE BONT J, MáRQUEZ S, FERNáNDEZ-BARRéS S, et al. Urban environment and obesity and weight-related behaviours in primary school children [J]. Environment international, 2021, 155: 106700.
[39] DENG M, LIU Y, MEI H, et al. Present situation investigation and intervention research of obesity in preschool children in Shunde district [J]. China Modern Medicine, 2013, 20(33): 165-6.
[40] DING X, HONG P, XU K, et al. Investigation on overweight and obesity of 3-6-year-old preschool children in Nanjing and analysis on the influencing factors [J]. Maternal and Child Health Care of China, 2019, 34(14): 3293-6.
[41] GUI Z H, ZHU Y N, CAI L, et al. Sugar-Sweetened Beverage Consumption and Risks of Obesity and Hypertension in Chinese Children and Adolescents: A National Cross-Sectional Analysis [J]. Nutrients, 2017, 9(12).
[42] HABOUSH-DELOYE A, BERLIN H, MARQUEZ E, et al. Obesity in Early Childhood: Examining the Relationship among Demographic, Behavioral, Nutritional, and

Socioeconomic Factors [J]. Childhood obesity (Print), 2021, 17(5): 349-56.
[43] HATAMI M, TAIB M N, JAMALUDDIN R, et al. Dietary factors as the major determinants of overweight and obesity among Iranian adolescents. A cross-sectional study [J]. Appetite, 2014, 82: 194-201.
[44] HEO M, WYLIE-ROSETT J. Being obese versus trying to lose weight: Relationship with physical inactivity and soda drinking among high school students [J]. The Journal of school health, 2020, 90(4): 301-5.
[45] HWANG S B, PARK S, JIN G R, et al. Trends in Beverage Consumption and Related Demographic Factors and Obesity among Korean Children and Adolescents [J]. Nutrients, 2020, 12(9).
[46] KARKI A, SHRESTHA A, SUBEDI N. Prevalence and associated factors of childhood overweight/obesity among primary school children in urban Nepal [J]. BMC public health, 2019, 19(1): 1055.
[47] LEON GUERRERO R T, BARBER L R, AFLAGUE T F, et al. Prevalence and Predictors of Overweight and Obesity among Young Children in the Children's Healthy Living Study on Guam [J]. Nutrients, 2020, 12(9).
[48] LIM S, ZOELLNER J M, LEE J M, et al. Obesity and sugar-sweetened beverages in African-American preschool children: a longitudinal study [J]. Obesity (Silver Spring, Md), 2009, 17(6): 1262-8.
[49] LIU J H, JONES S J, SUN H, et al. Diet, physical activity, and sedentary behaviors as risk factors for childhood obesity: an urban and rural comparison [J]. Childhood obesity (Print), 2012, 8(5): 440-8.
[50] LI S, ZHANG X, ZHANG M, et al. Influencing factors of overweight/obesity in children aged 3 to 6 in Xi’an City [J]. Chinese Journal of Woman and Child Health Research, 2016, 27(06): 681-4.
[51] LI S, ZHANG X, WANG S, et al. Analyses of the epidemiological ststus and the prevalence changes in recent 17 years of overweight and obesity in Xi'an children aged 0-7 years old [J]. Chinese Journal of Child Health Care, 2016, 24(10): 1044-7+51.
[52] LI S, WANG S, ZHANG S, et al. Investigation and influencing factors of overweight/obesity among children under 7 years old in Xi'an city, 2013 [J]. Chinese Journal of Child Health Care, 2017, 24(09): 924-7.
[53] LIU L, HUANG J, ZHOU Q, et al. Study on obesity ststus quo and influencing factors among school-age childre in hengde area [J]. Chongqing Medicine, 2017, 46(16): 2243-4+8.
[54] LIU Z, XU K, SUN Z, et al. Study on the current situation and risk factors of overweight and obesity among primary and secondary school students [J]. Chinese Journal of Prevention and Control of Chronic Diseases, 2020, 28(10): 743-7.
[55] MARTINEZ-OSPINA A, SUDFELD C R, GONZáLEZ S A, et al. School Food Environment, Food Consumption, and Indicators of Adiposity Among Students 7-14 Years in Bogotá, Colombia [J]. The Journal of school health, 2019, 89(3): 200-9.
[56] NASREDDINE L, NAJA F, AKL C, et al. Dietary, lifestyle and socio-economic correlates of overweight, obesity and central adiposity in Lebanese children and adolescents [J]. Nutrients, 2014, 6(3): 1038-62.
[57] OCHOA M C, MORENO-ALIAGA M J, MARTíNEZ-GONZáLEZ M A, et al. Predictor factors for childhood obesity in a Spanish case-control study [J]. Nutrition (Burbank, Los Angeles County, Calif), 2007, 23(5): 379-84.
[58] PAPANDREOU D, MALINDRETOS P, ROUSSO I. Risk factors for childhood obesity in a Greek paediatric population [J]. Public health nutrition, 2010, 13(10): 1535-9.
[59] PAYAB M, KELISHADI R, QORBANI M, et al. Association of junk food consumption with high blood pressure and obesity in Iranian children and adolescents: the CASPIAN-IV Study [J]. Jornal de pediatria, 2015, 91(2): 196-205.

[60] PENG R, LIU Y. Survey of obesity and the effect of comprehensive intervention in primary and secondary school students in Chengdu City [J]. Journal of Public Health and Preventive Medicine, 2020, 31(01): 109-12.
[61] QUAH P L, KLEIJWEG J, CHANG Y Y, et al. Association of sugar-sweetened beverage intake at 18 months and 5 years of age with adiposity outcomes at 6 years of age: the Singapore GUSTO mother-offspring cohort [J]. The British journal of nutrition, 2019, 122(11): 1303-12.
[62] RONG F, HUANG P, SONG H, et al. Overweight and obesity status and influencing factors in preschool children in Shanghai [J]. Journal of Mathematical Medicine, 2018, 31(01): 51-4.
[63] SAKAKI J R, MELOUGH M M, LI J, et al. Associations between Orange Juice Consumption and Dietary, Lifestyle and Anthropometric Characteristics in a Cross-Sectional Study of U.S. Children and Adolescents [J]. Nutrients, 2019, 11(11).
[64] SHEN T, HE P, LI L, et al. Investigation on the correlation between diet and obesity among preschool children in Shanghai, Wuxi and Kunsha [J]. Maternal and Child Health Care of China, 2014, 29(21): 3471-4.
[65] SONG Y, DENG Q, YANG M, et al. The correlation between beverage consumption behavior and overweight and obesity among middle school students in Shangrao [J]. Chinese Journal of School Health, 2020, 41(04): 591-4.
[66] SHAN X Y, XI B, CHENG H, et al. Prevalence and behavioral risk factors of overweight and obesity among children aged 2-18 in Beijing, China [J]. International journal of pediatric obesity : IJPO : an official journal of the International Association for the Study of Obesity, 2010, 5(5): 383-9.
[67] TAN X, LIU D, XU L. Influencing factors of childhood overweight and obesity based on 5-2-1-0 model [J]. Chinese Journal of Child Health Care, 2012, 20(02): 127-9.
[68] VALENTE H, TEIXEIRA V, PADRãO P, et al. Sugar-sweetened beverage intake and overweight in children from a Mediterranean country [J]. Public health nutrition, 2011, 14(1): 127-32.
[69] VINCIGUERRA F, TUMMINIA A, ROPPOLO F, et al. Impact of unhealthy childhood and unfavorable parents' characteristics on adiposity in schoolchildren [J]. Diabetes/metabolism research and reviews, 2019, 35(8): e3199.
[70] WANG H, JEONG H, KIM N H, et al. Association between beverage intake and obesity in children: The Korea National Health and Nutrition Examination Survey (KNHANES) 2013-2015 [J]. Nutrition research and practice, 2018, 12(4): 307-14.
[71] WANG J, SUN Y, SONG W, et al. Study on the relationship between sugar-sweetened beverage intake and central obesity in children [J]. Chinese journal of Disease Control & Prevention, 2021, 25(05): 534-40.
[72] WU Y, LIU Y, XIANG M, et al. Association between dietary behaviors and overweight or obesity in preschool children [J]. Journal of Environmental & Occupational Medicine, 2022, 39(06): 672-8.
[73] YU J, HUANG F, ZHANG X, et al. Association of Sugar-Sweetened Beverage Consumption and Moderate-to-Vigorous Physical Activity with Childhood and Adolescent Overweight/Obesity: Findings from a Surveillance Project in Jiangsu Province of China [J]. Nutrients, 2023, 15(19).
[74] ZHANG J, ZHANG J, YANG J, et al. Obesity and influencing factoe among primary and secondary school students in Jiangsu Province [J]. Chinese Journal of School Health, 2019, 40(05): 778-80.
[75] ZHANG J. Analysis of the incidence of overweight and obesity and related influencing factors in children aged 0-6 years in Chaoyang District of Beijing [D]; Bejiing University of Chinese Medicine, 2018.
[76] ANDEGIORGISH A K, WANG J, ZHANG X, et al. Prevalence of overweight, obesity, and associated risk factors among school children and adolescents in Tianjin, China [J]. Eur J Pediatr, 2012, 171(4): 697-703.

[77] ANDROUTSOS O, MOSCHONIS G, IERODIAKONOU D, et al. Perinatal and lifestyle factors mediate the association between maternal education and preschool children's weight status: the ToyBox study [J]. Nutrition, 2018, 48: 6-12.
[78] BHUIYAN M U, ZAMAN S, AHMED T. Risk factors associated with overweight and obesity among urban school children and adolescents in Bangladesh: a case-control study [J]. BMC Pediatr, 2013, 13: 72.
[79] BIBILONI MDEL M, MARTINEZ E, LLULL R, et al. Prevalence and risk factors for obesity in Balearic Islands adolescents [J]. Br J Nutr, 2010, 103(1): 99-106.
[80] BROPHY S, COOKSEY R, GRAVENOR M B, et al. Risk factors for childhood obesity at age 5: analysis of the millennium cohort study [J]. BMC Public Health, 2009, 9: 467.
[81] CHEN Y C, CHEN P C, HSIEH W S, et al. Environmental factors associated with overweight and obesity in taiwanese children [J]. Paediatr Perinat Epidemiol, 2012, 26(6): 561-71.
[82] CHEN J, LUO S, LIANG X, et al. The relationship between socioeconomic status and childhood overweight/obesity is linked through paternal obesity and dietary intake: a cross-sectional study in Chongqing, China [J]. Environ Health Prev Med, 2021, 26(1): 56.
[83] COOK W K, TSENG W. Associations of Asian Ethnicity and Parental Education with Overweight in Asian American Children and Adolescents: An Analysis of 2011-2016 National Health and Nutrition Examination Surveys [J]. Matern Child Health J, 2019, 23(4): 504-11.
[84] DING S, CHEN J, DONG B, et al. Association between parental socioeconomic status and offspring overweight/obesity from the China Family Panel Studies: a longitudinal survey [J]. BMJ Open, 2021, 11(4): e045433.
[85] DONKOR H M, GRUNDT J H, JULIUSSON P B, et al. Social and somatic determinants of underweight, overweight and obesity at 5 years of age: a Norwegian regional cohort study [J]. BMJ Open, 2017, 7(8): e014548.
[86] FENG Y, DING L, TANG X, et al. Association between Maternal Education and School-Age Children Weight Status: A Study from the China Health Nutrition Survey, 2011 [J]. Int J Environ Res Public Health, 2019, 16(14): 2543.
[87] FRYE C, HEINRICH J. Trends and predictors of overweight and obesity in East German children [J]. Int J Obes Relat Metab Disord, 2003, 27(8): 963-9.
[88] FUIANO N, RAPA A, MONZANI A, et al. Prevalence and risk factors for overweight and obesity in a population of Italian schoolchildren: a longitudinal study [J]. J Endocrinol Invest, 2008, 31(11): 979-84.
[89] GRYDELAND M, BERGH I H, BJELLAND M, et al. Correlates of weight status among Norwegian 11-year-olds: The HEIA study [J]. BMC Public Health, 2012, 12: 1053.
[90] GURZKOWSKA B, KULAGA Z, LITWIN M, et al. The relationship between selected socioeconomic factors and basic anthropometric parameters of school-aged children and adolescents in Poland [J]. Eur J Pediatr, 2014, 173(1): 45-52.
[91] GAO M, WELLS J C K, JOHNSON W, et al. Socio-economic disparities in child-to-adolescent growth trajectories in China: Findings from the China Health and Nutrition Survey 1991-2015 [J]. The Lancet regional health Western Pacific, 2022, 21: 100399.
[92] HAAS J S, LEE L B, KAPLAN C P, et al. The association of race, socioeconomic status, and health insurance status with the prevalence of overweight among children and adolescents [J]. Am J Public Health, 2003, 93(12): 2105-10.
[93] HERTER-AEBERLI I, OSUNA E, SARNOVSKA Z, et al. Significant Decrease in Childhood Obesity and Waist Circumference over 15 Years in Switzerland: A Repeated Cross-Sectional Study [J]. Nutrients, 2019, 11(8).
[94] HOANG N T D, ORELLANA L, LE T D, et al. Anthropometric Status among 6(-)9-Year-Old School Children in Rural Areas in Hai Phong City, Vietnam [J]. Nutrients, 2018, 10(10).
[95] HOMS C, BERRUEZO P, ARCARONS A, et al. Independent and Joined Association between Socioeconomic Indicators and Pediatric Obesity in Spain: The PASOS Study [J].

Nutrients, 2023, 15(8).
[96] IKEDA N, NISHI N. First incidence and associated factors of overweight and obesity from preschool to primary school: longitudinal analysis of a national cohort in Japan [J]. Int J Obes (Lond), 2019, 43(4): 751-60.
[97] IP P, HO F K, SO H K, et al. Socioeconomic Gradient in Childhood Obesity and Hypertension: A Multilevel Population-Based Study in a Chinese Community [J]. PLoS One, 2016, 11(6): e0156945.
[98] INOUE K, SEEMAN T E, NIANOGO R, et al. The effect of poverty on the relationship between household education levels and obesity in U.S. children and adolescents: an observational study [J]. Lancet regional health Americas, 2023, 25: 100565.
[99] JULIUSSON P B, EIDE G E, ROELANTS M, et al. Overweight and obesity in Norwegian children: prevalence and socio-demographic risk factors [J]. Acta Paediatr, 2010, 99(6): 900-5.
[100] KEANE E, LAYTE R, HARRINGTON J, et al. Measured parental weight status and familial socio-economic status correlates with childhood overweight and obesity at age 9 [J]. PLoS One, 2012, 7(8): e43503.
[101] KLEIN-PLATAT C, WAGNER A, HAAN M C, et al. Prevalence and sociodemographic determinants of overweight in young French adolescents [J]. Diabetes Metab Res Rev, 2003, 19(2): 153-8.
[102] KE Y, ZHANG S, HAO Y, et al. Associations between socioeconomic status and risk of obesity and overweight among Chinese children and adolescents [J]. BMC public health, 2023, 23(1): 401.
[103] LAMERZ A, KUEPPER-NYBELEN J, WEHLE C, et al. Social class, parental education, and obesity prevalence in a study of six-year-old children in Germany [J]. Int J Obes (Lond), 2005, 29(4): 373-80.
[104] LASSERRE A M, CHIOLERO A, CACHAT F, et al. Overweight in Swiss children and associations with children's and parents' characteristics [J]. Obesity (Silver Spring), 2007, 15(12): 2912-9.
[105] LAZZERI G, GIACCHI M V, SPINELLI A, et al. Overweight among students aged 11-15 years and its relationship with breakfast, area of residence and parents' education: results from the Italian HBSC 2010 cross-sectional study [J]. Nutr J, 2014, 13: 69.
[106] LE G B, DINH D X. Prevalence and associated factors of overweight and obesity among primary school children: a cross-sectional study in Thanhhoa City, Vietnam [J]. BMJ Open, 2022, 12(4): e058504.
[107] LEE H J, KIM S H, JIN M H, et al. Variability in sociodemographic factors and obesity in Korean children: a cross-sectional analysis of Korea National Health and Nutrition Examination survey data (2007-2015) [J]. Ann Epidemiol, 2020, 43: 51-7.
[108] LI M, LIU Y, XU P, et al. Obesity, overweight and related factors of Kazakh children aged from 6 to 13 in Yili, Xinjiang [J]. Chin J Pre Med, 2011, 45(6): 506-11.
[109] LI Q, YU Z, LIN R, et al. Analysis on growth and development level and influencing factors of preschool children in Qingcheng District, Qingyuan City [J]. Yi Xue Shi Liao Yu Jian Kang, 2021, 19(14): 7-9.
[110] LIANG R, YANG L, YANG H, et al. Analysis of the influencing factors for overweight/obesity of 3 to 6-year-old children in Pudong New Area， Shanghai [J]. CJCHC DEC, 2018, 25(12): 1304-7.
[111] LIU W, LIU W, LIN R, et al. Socioeconomic determinants of childhood obesity among primary school children in Guangzhou, China [J]. BMC Public Health, 2016, 16: 482.
[112] LIU L, MA Y, JIANG N, et al. Interaction between Parental Education and Household Wealth on Children’s Obesity Risk [J]. Int J Environ Res Public Health, 2018, 15(8): 1754.
[113] LOMBARDO F L, SPINELLI A, LAZZERI G, et al. Severe obesity prevalence in 8- to 9-year-old Italian children: a large population-based study [J]. Eur J Clin Nutr, 2015, 69(5):

603-8.
[114] MAZUR A, KLIMEK K, TELEGA G, et al. Risk factors for obesity development in school children from south-eastern Poland [J]. Ann Agric Environ Med, 2008, 15(2): 281-5.
[115] MUSIC MILANOVIC S, LANG MOROVIC M, BUKAL D, et al. Regional and sociodemographic determinants of the prevalence of overweight and obesity in children aged 7-9 years in Croatia [J]. Acta Clin Croat, 2020, 59(2): 303-11.
[116] MORAEUS L, LISSNER L, YNGVE A, et al. Multi-level influences on childhood obesity in Sweden: societal factors, parental determinants and child's lifestyle [J]. Int J Obes (Lond), 2012, 36(7): 969-76.
[117] MOSCHONIS G, SIOPIS G, ANASTASIOU C, et al. Prevalence of Childhood Obesity by Country, Family Socio-Demographics, and Parental Obesity in Europe: The Feel4Diabetes Study [J]. Nutrients, 2022, 14(9).
[118] MOSCHONIS G, TANAGRA S, VANDOROU A, et al. Social, economic and demographic correlates of overweight and obesity in primary-school children: preliminary data from the Healthy Growth Study [J]. Public Health Nutr, 2010, 13(10A): 1693-700.
[119] MURER S B, SAARSALU S, ZIMMERMANN J, et al. Risk factors for overweight and obesity in Swiss primary school children: results from a representative national survey [J]. Eur J Nutr, 2016, 55(2): 621-9.
[120] NOH J W, KIM Y E, PARK J, et al. Impact of parental socioeconomic status on childhood and adolescent overweight and underweight in Korea [J]. J Epidemiol, 2014, 24(3): 221-9.
[121] NOH J W, KIM Y E, OH I H, et al. Influences of socioeconomic factors on childhood and adolescent overweight by gender in Korea: cross-sectional analysis of nationally representative sample [J]. BMC Public Health, 2014, 14: 324.
[122] PADEZ C, MOURAO I, MOREIRA P, et al. Prevalence and risk factors for overweight and obesity in Portuguese children [J]. Acta Paediatr, 2005, 94(11): 1550-7.
[123] PADUANO S, BORSARI L, SALVIA C, et al. Risk Factors for Overweight and Obesity in Children Attending the First Year of Primary Schools in Modena, Italy [J]. J Community Health, 2020, 45(2): 301-9.
[124] PATSOPOULOU A, TSIMTSIOU Z, KATSIOULIS A, et al. Prevalence and Risk Factors of Overweight and Obesity among Adolescents and Their Parents in Central Greece (FETA Project) [J]. Int J Environ Res Public Health, 2015, 13(1): 83.
[125] PHAM T T P, MATSUSHITA Y, DINH L T K, et al. Prevalence and associated factors of overweight and obesity among schoolchildren in Hanoi, Vietnam [J]. BMC Public Health, 2019, 19(1): 1478.
[126] SAHA J, CHOUHAN P, AHMED F, et al. Overweight/Obesity Prevalence among Under-Five Children and Risk Factors in India: A Cross-Sectional Study Using the National Family Health Survey (2015-2016) [J]. Nutrients, 2022, 14(17).
[127] SANTIAGO S, ZAZPE I, CUERVO M, et al. Perinatal and parental determinants of childhood overweight in 6-12 years old children [J]. Nutr Hosp, 2012, 27(2): 599-605.
[128] SCHOOLING C M, YAU C, COWLING B J, et al. Socio-economic disparities of childhood Body Mass Index in a newly developed population: evidence from Hong Kong's 'Children of 1997' birth cohort [J]. Arch Dis Child, 2010, 95(6): 437-43.
[129] SCHULE S A, FROMME H, BOLTE G. Built and socioeconomic neighbourhood environments and overweight in preschool aged children. A multilevel study to disentangle individual and contextual relationships [J]. Environ Res, 2016, 150: 328-36.
[130] SHE T. Correlation Analysis of Social Economic Factors of Overweight and Obesity Children in China [J]. Journal of Guangzhou Sport University, 2015, 35(35-38).
[131] SINGH G K, KOGAN M D, VAN DYCK P C, et al. Racial/ethnic, socioeconomic, and behavioral determinants of childhood and adolescent obesity in the United States: analyzing independent and joint associations [J]. Ann Epidemiol, 2008, 18(9): 682-95.

[132] SUN G, JIA G, PENG H, et al. Trends of childhood obesity in China and associated factors [J]. Clin Nurs Res, 2015, 24(2): 156-71.
[133] VELDHUIS L, VOGEL I, VAN ROSSEM L, et al. Influence of maternal and child lifestyle-related characteristics on the socioeconomic inequality in overweight and obesity among 5-year-old children; the "Be Active, Eat Right" Study [J]. Int J Environ Res Public Health, 2013, 10(6): 2336-47.
[134] VELTSISTA A, KANAKA C, GIKA A, et al. Tracking of overweight and obesity in Greek youth [J]. Obes Facts, 2010, 3(3): 166-72.
[135] WANG J J, GAO Y, LAU P W C. Prevalence of overweight in Hong Kong Chinese children: Its associations with family, early-life development and behaviors-related factors [J]. J Exerc Sci Fit, 2017, 15(2): 89-95.
[136] WU X, WANG Y, FAN L, et al. Prevalence and risk factors of obesity among children aged 7-15 years in Pudong, China [J]. Prev Med, 2018, 30(10): 1060-3.
[137] XU X, PAN C-L, LIU G-L, et al. Socioeconomic and lifestyle behavioral factors associated with overweight and obesity among rural to urban migrant children in central China [J]. Int J Clin Exp Med, 2016, 9(11): 21635-44.
[138] YI X, YIN C, CHANG M, et al. Prevalence and risk factors of obesity among school-aged children in Xi'an, China [J]. Eur J Pediatr, 2012, 171(2): 389-94.
[139] ZHAI J, XUE H, LUO J, et al. Associations between socioeconomic status and overweight among urban children aged 7-12 years in Chengdu, southwest China [J]. Asia Pac J Clin Nutr, 2018, 27(3): 617-23.
[140] ZHANG Y, LI J, WAN J, et al. Prevalence and risk factors of overweight and obesity among children in Nanchang, China [J]. Chin J Sch Health, 2015, 36(8).
[141] DENG Z, MAO G, WANG Y, et al. Evaluation of nutritional status of school-age children after implementation of "Nutrition Improvement Program" in rural area in Hunan, China [J]. Chin J Contemp Pediatr, 2016, 18(9): 851-6.
[142] DING Y, MA L, JIA P, et al. Longitudinal effects of school policies on children's eating behaviors and weight status: findings from the childhood obesity study in China megacities [J]. International journal of obesity (2005), 2023, 47(1): 17-23.
[143] FU Y C A, TO K C, TAO W Y, et al. School accreditation scheme reduces childhood obesity in Hong Kong [J]. Glob Health Promot, 2019, 26(4): 70-8.
[144] KENNEY E L, BARRETT J L, BLEICH S N, et al. Impact Of The Healthy, Hunger-Free Kids Act On Obesity Trends [J]. Health Aff (Millwood), 2020, 39(7): 1122-9.
[145] LEUNG C W, BLUMENTHAL S J, HOFFNAGLE E E, et al. Associations of food stamp participation with dietary quality and obesity in children [J]. Pediatrics, 2013, 131(3): 463-72.
[146] LI L, DUAN R, XIE L, et al. Evaluation of "Nutrition Improvement Program" for rural boarding studentst in Baoshan, China 2015 [J]. China Health Care & Nutrition, 2016, 26: 44-5.
[147] ØVREBø B, STEA T H, BERGH I H, et al. A nationwide school fruit and vegetable policy and childhood and adolescent overweight: A quasi-natural experimental study [J]. PLoS medicine, 2022, 19(1): e1003881.
[148] RITO A I, CARVALHO M A, RAMOS C, et al. Program Obesity Zero (POZ) – a community-based intervention to address overweight primary-school children from five Portuguese municipalities [J]. Public Health Nutr, 2013, 16(6): 1043-51.
[149] SEKHOBO J P, EDMUNDS L S, REYNOLDS D K, et al. Trends in Prevalence of Obesity and Overweight Among Children Enrolled in the New York State WIC Program, 2002–2007 [J]. Public Health Rep, 2010, 125(2): 218-24.
[150] SEKHOBO J P, EDMUNDS L S, DALENIUS K, et al. Neighborhood disparities in prevalence of childhood obesity among low-income children before and after implementation of New York City child care regulations [J]. Prev Chronic Dis, 2014, 11: E181.
[151] SEO D C, LEE C G. Association of school nutrition policy and parental control with childhood overweight [J]. J Sch Health, 2012, 82(6): 285-93.
[152] SIRIKULCHAYANONTA C, SIRIKULCHAYANONTA V, SURIYAPROM K, et al. Changing trends of obesity and lipid profiles among Bangkok school children after

comprehensive management of the bright and healthy Thai kid project [J]. BMC Public Health, 2022, 22(1): 1323.
[153] KREIDER B, PEPPER J V, GUNDERSEN C, et al. Identifying the Effects of SNAP (Food Stamps) on Child Health Outcomes When Participation Is Endogenous and Misreported [J]. Journal of the American Statistical Association, 2012, 107(499): 958-75.
[154] SIMMONS S, ALEXANDER J L, EWING H, et al. SNAP Participation in Preschool-Aged Children and Prevalence of Overweight and Obesity [J]. 2012, 82(12): 548-52.

Supplemental table 4. Economic characteristic of included studies

Study	Country/region	Time [1]	GNI per capita	
			Value ($)	Classification
Bel-Serrat (2019) [1]	Ireland	2015	47665.73	HIC
Chang (2012) [1]	China-Liaoning	2010	1748.53	L&MIC
Chen (2007) [1]	China-Guangdong	2005	1282.26	L&MIC
Chen (2017) [1]	China-Guangdong	2015	3641.62	L&MIC
Chen (2021) [1]	China-Jiangxi	2020	4371.74	L&MIC
Dudas (2008) [1]	USA	2006	54280.23	HIC
Dupuy (2011) [1]	France	2009	35752.86	HIC
Flores (2013) [1]	USA	2001	49425.52	HIC
Guan (2018) [1]	China-Guangdong	2015	3641.62	L&MIC
Guo (2011) [1]	China-Hubei	2010	1586.54	L&MIC
Hong (2010) [1]	Vietnam	2004 (GNI2015)	2450.63	L&MIC
Huang (2017) [1]	China-Sichuan	2015	2756.17	L&MIC
Jia (2013) [1]	China-Zhejiang	2010	2745.79	L&MIC
Kolmaga (2019) [1]	Poland	2012	11099.65	L&MIC
Li (2021) [1]	China-Fujian	2020	5352.95	L&MIC
Lu (2018) [1]	China-Beijing	2015	5553.39	L&MIC
Luo (2020) [1]	China-Henan	2020	3981.41	L&MIC
Nie (2014) [1]	China-Qinghai	2010	1244.19	L&MIC
Pengpid (2016) [1]	Southeast Asian Nations (ASEAN)	2013	1369.69	L&MIC
Qin (2013) [1]	China-Chongqing	2010	1619.72	L&MIC
Qiu (2020) [1]	China-Henan	2020	3981.41	L&MIC
Salahuddin (2017) [1]	USA (Low-Income, Predominantly Hispanic/Latino)	2014	56846.8	HIC
Santiago (2013) [1]	Spain	2011	24964.65	HIC
Wang (2016) [1]	China	2015	7978.18	L&MIC
Wei (2009) [1]	China-Guangdong	2005	1282.26	L&MIC
Wu (2019) [1]	China-Shanghai	2015	5721.61	L&MIC
Xiang (2011) [1]	China-Qinghai	2010	1244.19	L&MIC
Yang (2021) [1]	China-Inner Mongolia	2020	4604.43	L&MIC
Zeng (2010) [1]	China-Jilin	2010	1537.5	L&MIC
Zhang (2011) [1]	China-Shanghai	2005	1807.16	L&MIC
Zhang (2013)-1 [1]	China-Shandong	2010	1846.81	L&MIC
Zhang (2013)-2 [1]	China-Chongqing	2010	1619.72	L&MIC
Zou (2020) [1]	China-Hubei	2020	4169.15	L&MIC
Zurriaga (2011) [1]	Span	2009	25743.02	HIC
Beck (2014) [2]	USA	2010	49150	HIC

Calvo (2014)[2]	Spain	2001	24150.27	HIC
Cao (2008)[2]	China-Xinjiang	2005	689.17	L&MIC
Chang (2012)[2]	China-Liaoning	2010	1748.53	L&MIC
de Bont (2021)[2]	Spain	2017-2019	29330	HIC
Deng (2013)[2]	China-Guangdong	2010	2257.4	L&MIC
Ding (2019)[2]	China-Jiangsu	2015	4014.06	L&MIC
Dudas (2008)[2]	USA	2006	54280.23	HIC
Flores (2013)[2]	USA	2001	49425.52	HIC
Guan (2018)[2]	China-Guangdong	2015	3641.62	L&MIC
Gui (2017)[2]	China	2015	7978.18	L&MIC
Haboush-Deloye (2021)[2]	USA	2012	52790	HIC
Hatami (2014)[2]	Iran	2009-2010	5450	L&MIC
Heo (2020)[2]	USA	2013	53990	HIC
Hwang (2020)[2]	Korea	2018	32750	HIC
Hong (2010)[2]	Vietnam	2004 (GNI2015)	2450.63	L&MIC
Karki (2019)[2]	Nepal	2017	975.19	L&MIC
Leon-Guerrero (2020)[2]	USA	2013	53990	HIC
Lim (2009)[2]	USA	2002-2005	37310	HIC
Liu (2012)[2]	USA	2006	47830	HIC
Li (2016)-1[2]	China-Shaanxi	2015	2679.18	L&MIC
Li (2016)-2[2]	China-Shaanxi	2015	2679.18	L&MIC
Li (2017)[2]	China-Shaanxi	2015	2679.18	L&MIC
Li (2021)[2]	China-Fujian	2020	5352.95	L&MIC
Liu (2020)[2]	China-Hebei	2015	2800.55	L&MIC
Liu (2018)[2]	China-Tianjin	2020	5645.12	L&MIC
Martinez-Ospina (2019)[2]	USA	2015	56620	HIC
Nasreddine (2014)[2]	Lebanon	2009	6680	L&MIC
Ochoa (2007)[2]	Spain	2004	22130	HIC
Papandreou (2010)[2]	Greek	2007	26370	HIC
Payab (2015)[2]	Iran	2011-2012	6860	L&MIC
Peng (2020)[2]	China-Sichuan	2020	4290.26	L&MIC
Pengpid (2016)[2]	Southeast Asian Nations (ASEAN)	2016	1628.38	L&MIC
Quah (2019)[2]	Singapore	2009	37320	HIC
Rong (2018)[2]	China-Shanghai	2015	5721.61	L&MIC
Sakaki (2019)[2]	USA	2004	51733.44	HIC
Shan (2010)[2]	China Beijing	2007	3160.32	L&MIC
Shen (2014)[2]	China-Shanghai, Wuxi, Kunshan	2010	3256.09	L&MIC
Song (2020)[2]	China-Jiangxi	2020	4371.74	L&MIC
Tan (2012)[2]	China-Shandong	2010	1846.81	L&MIC

Valente (2011) [2]	Portugal	2007	20860	HIC
Vinciguerra (2019) [2]	Italy	2014	34910	HIC
Wang (2021) [2]	China	2010	5622.76	L&MIC
Wei (2009) [2]	China-Guangdong	2005	1282.26	L&MIC
Wu (2022) [2]	China-Shanghai	2015	5721.61	L&MIC
Yu (2023) [2]	China-Jiangsu	2020	6062.66	L&MIC
Zhang (2018) [2]	China-Beijing	2015	5553.39	L&MIC
Zhang (2019)[2]	China-Jiangsu	2015	4014.06	L&MIC
Zurriaga (2011) [2]	Span	2008	26446.3	L&MIC
Andegiorgish (2012) [3]	China-Tianjin	2010	2241.77	L&MIC
Androutsos (2018) [3]	Europe	2012	29286.2	HIC
Bhuiyan (2013) [3]	Bangladesh	2007	913.36	L&MIC
Bibioni (2010) [3]	Spain	2007-2008	26446.3	HIC
Brophy (2009) [3]	UK	2004	41962	HIC
Chen (2012) [3]	China-Taiwan	2007-2010	8800.49	L&MIC
Chen (2021) [3]	China-Chongqing	2014	2856.01	L&MIC
Cook (2019) [3]	USA	2011-2016	58460.3	HIC
Ding (2021) [3]	China	2010-2016	7978.18	L&MIC
Donkor (2017) [3]	Norway	2001-2007	78135.4	HIC
Feng (2019) [3]	China	2011	5622.76	L&MIC
Frye (2003) [3]	German	1992-1999	33589.2	HIC
Fuiano (2008) [3]	Italy	2005-2007	34032.2	HIC
Grydeland (2012) [3]	Norway	2007	78135.4	HIC
Gurzkowska (2014) [3]	Poland	2010	10476.3	L&MIC
Gao (2022) [3]	China	1996	650	L&MIC
Haas (2003) [3]	USA	1996	42438.4	HIC
Herter-Aeberli (2019) [3]	Switzerland	2002-2018	82603.5	HIC
Homs (2023) [3]	Spain	2019	30360	HIC
Hoang (2018) [3]	Vietnam	2016	2450.63	L&MIC
Ikeda (2019) [3]	Japan	2001-2011	33723.1	HIC
Ip (2016) [3]	China-Hong Kong	2005-2006	33837.1	HIC
Inoue (2023) [3]	USA	2010	49150	HIC
Juliusson (2010) [3]	Norway	2012	77771.9	HIC
Keane (2012) [3]	Ireland	2009	38874.9	HIC
Klein-Platat (2003) [3]	France	2001	34393.2	HIC
Ke (2023) [3]	China-Jiangsu, Anhui, Zhejiang, and Shanghai	2020	6639.5	Low
Lamerz (2005) [3]	German	2001-2002	34531.6	HIC
Lasserre (2007) [3]	Switzerland	2005-2006	84696.6	HIC
Lazzeri (2014) [3]	Italy	2010	31809.8	HIC
Le (2022) [3]	Vietnam	2021	2450.63	L&MIC

Lee (2020) [3]	Korea	2007-2015	28822.1	HIC
Li (2011) [3]	China-Xinjiang	2004-2007	1298.65	L&MIC
Li (2021) [3]	China-Guangdong	2016	3641.62	L&MIC
Liang (2018) [3]	China-Shanghai	2018	8730.9	L&MIC
Liu (2016) [3]	China-Guangdong	2014	3641.62	L&MIC
Liu (2018) [3]	China-Liaoning	2017	3266.74	L&MIC
Lombardo (2015) [3]	Italy	2010	31809.8	HIC
Mazur (2008) [3]	Poland	2003	7804.67	L&MIC
Milanovic (2020) [3]	Croatia	2015-2016	12197.8	L&MIC
Moraeus (2012) [3]	Sweden	2008	50292.7	HIC
Moschonis (2010) [3]	Greece	2007	22985	HIC
Moschonis (2022) [3]	Europe	2016-2018	32530.1	HIC
Murer (2016) [3]	Switzerland	2011	83863.1	HIC
Noh (2014)-1[3]	Korea	2009	23273.2	HIC
Noh (2014)-2[3]	Korea	2009	23273.2	HIC
Padez (2005) [3]	Portugal	2002-2003	18207.6	HIC
Paduano (2020) [3]	Italy	2018	32036.4	HIC
Patsopoulou (2015) [3]	Greece	2011	18479.7	HIC
Pham (2019) [3]	Vietnam	2014	2450.63	L&MIC
Saha (2022) [3]	India	2015-2016	1699.58	L&MIC
Santiago (2012) [3]	Spain	2008	26446.3	HIC
Schooling (2010) [3]	China-Hong Kong	1997-2008	38725.9	HIC
Schule (2016) [3]	German	2004-2007	38193.3	HIC
She (2015) [3]	China-Sichuan	2010	1462.93	L&MIC
Singh (2008) [3]	USA	2003	50303.7	HIC
Sun (2015) [3]	China-Jiangsu	2004-2007	1178.48	L&MIC
Veldhuis (2013) [3]	Netherlands	2007-2008	31834.3	HIC
Veltsista (2010) [3]	Greece	1990, 2001	22429.4	HIC
Wang (2017) [3]	China-Hong Kong	2015	43217	HIC
Wu (2018) [3]	China-Shanghai	2016-2017	5721.61	L&MIC
Xu (2016) [3]	China- Nanchang, Wuhan, and Changsha	2011	5622.76	L&MIC
Yi (2012) [3]	China-Shaanxi	2008-2009	1407.48	L&MIC
Zhai (2018) [3]	China-Sichuan	2013	2756.17	L&MIC
Zhang (2015) [3]	China-Jiangxi	2013	2834.26	L&MIC
Deng (2016) [4]	China-Hunan	2012-2015	3015.86	L&MIC
Ding (2023) [4]	China-Beijing, Shanghai, Nanjing, Chengdu, and Xi'an	2015	5721.61	L&MIC
Fu (2018) [4]	China-Hong Kong	2007-2009	37634.59	HIC
Kenney (2020) [4]	USA	2003-2018	61050.36	HIC
Leung (2012) [4]	USA	2002-2007	54144.87	HIC

Øvrebø (2022) [4]	Norway	2010	88940	HIC
Li (2016) [4]	China-Yunnan	2012-2015	2468.25	L&MIC
Rito (2013) [4]	Portugal	2003-2008	18709.62	HIC
Sekhobo (2010) [4]	USA	2003-2007	54144.87	HIC
Sekhobo (2014) [4]	USA	2004-2008	52734.11	HIC
Seo (2012) [4]	USA	2002-2007	54144.87	HIC
Sirikulchayanonta (2022) [4]	Thailand	2012-2014	5538.79	L&MIC
Kreider (2012) [4]	USA	2001-2006	51733.44	HIC
Simmons (2012) [4]	USA	2008-2010	49150	HIC

GNI, gross national income; HIC, high income country/region; L&MIC, low- and middle-income country/region

HIC: GNI per capita was more than $13,205; L&MIC: GNI per capita was less than $13,205.

[1] Economic characteristic of the 34 studies of the association between fruit and/or vegetable intake and overweight and/or obesity:

[2] Economic characteristic of the 50 studies of the association between sugar-sweetened beverage intake and overweight and/or obesity

[3] Economic characteristic of the 65 studies of the association between parental education level and overweight and/or obesity

[4] Economic characteristic of the 12 studies of the association between nutrition policy and overweight and/or obesity

Supplemental table 5. Association between diet intake, parental education and nutrition policy and children's health and development stratified by different Continent[1]

	N_{study}	OR (95% CI)	I^2
Fuit and/or vegetable intakes and risk of overweight and/or obesity			
High income			
Europe	4	0.87 (0.67, 1.14)	75.6%
America	3	0.54 (0.23, 1.26)	81.2%
Low- and middle income			
China	24	0.79 (0.71, 0.87)	90.0%
Other Asia	2	0.59 (0.16, 2.21)	96.4%
Other Europe (Poland)	1	0.54 (0.29, 0.99)	.
Beverage intakes and risk of overweight and/or obesity			
High income			
Europe	7	1.31 (0.98, 1.74)	79.3%
America	10	1.19 (1.08, 1.32)	80.0%
Other Asia	3	1.50 (1.00, 2.28)	67.7%
Low- and middle income			
China	24	1.31 (1.21, 1.42)	84.4%
Other Asia	6	1.25 (1.12, 1.40)	0.0%
Parental education level and risk of overweight and/or obesity			
High income			
Europe	27	1.49 (1.33, 1.68)	75.2%
America	11	1.36 (1.14, 1.60)	86.2%
Low- and middle income			
China	19	1.10 (0.89, 1.36)	90.6%
Other Asia	5	0.80 (0.50, 1.30)	89.2%
Other Europe (Poland and Croatia)	3	1.08 (0.94, 1.23)	18.2%
Nutrition policy implementation and risk of overweight and/or obesity			
High income			
Europe	2	0.97 (0.88, 1.07)	0.0%
America	7	0.96 (0.91, 1.02)	89.9%
China Hongkong	1	0.92 (0.85, 0.99)	.
Low- and middle-income			
China	3	1.03 (0.79, 1.33)	97.0%
Other Asia	1	0.69 (0.48, 1.00)	.

[1] High income: GNI per capita was more than $13,205;
Low- and middle-income: GNI per capita was less than $13,205.

Supplemental table 6. Association between nutrition policy and children's overweight and obesity stratified by different type nutrition policy[1]

	N_{study}	OR (95% CI)	I^2
High income			
Nutrition improvement program at the national or regional	2	0.88 (0.72, 1.07)	30.7%
School nutrition policy	8	0.97 (0.92, 1.02)	85.1%
Low- and middle income			
Nutrition improvement program at the national or regional level	1	0.33 (0.17, 0.64)	0.0%
School nutrition policy	3	1.07 (0.85, 1.34)	96.7%

[1] High income: GNI per capita was more than $13,205;
Low- and middle-income: GNI per capita was less than $13,205.

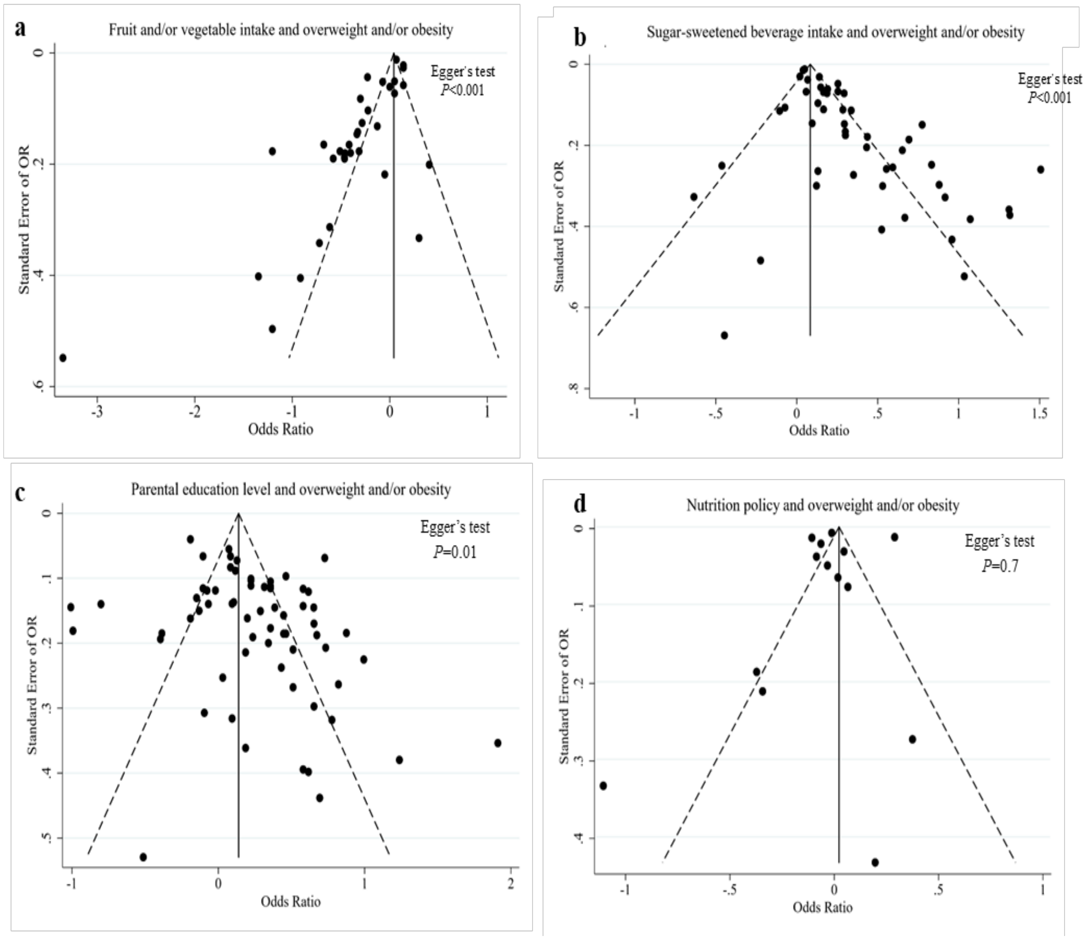

Supplemental figure 1. Funnel plot of the relative risks of studies

[a] 34 studies on fruit and/or vegetable intake and the risk of overweight and/obesity;

[b] 50 studies on sugar-sweetened beverage intake and risk of overweight and/or obesity;

[c] 65 studies on parental education level and the risk of overweight and/or obesity;

[d] 14 studies on nutrition policy and the risk of overweight and/or obesity;
